# Genome-wide identification and transcriptional expression analysis of mitogen-activated protein kinase and mitogen-activated protein kinase kinase genes in *Capsicum annuum*

**DOI:** 10.3389/fpls.2015.00780

**Published:** 2015-09-25

**Authors:** Zhiqin Liu, Lanping Shi, Yanyan Liu, Qian Tang, Lei Shen, Sheng Yang, Jinsen Cai, Huanxin Yu, Rongzhang Wang, Jiayu Wen, Youquan Lin, Jiong Hu, Cailing Liu, Yangwen Zhang, Shaoliang Mou, Shuilin He

**Affiliations:** ^1^National Education Minster Key Laboratory of Plant Genetic Improvement and Comprehensive Utilization, Fujian Agriculture and Forestry UniversityFuzhou, China; ^2^College of Plant Protection, Fujian Agriculture and Forestry UniversityFuzhou, China; ^3^College of Crop Science, Fujian Agriculture and Forestry UniversityFuzhou, China; ^4^College of Life Science, Fujian Agriculture and Forestry UniversityFuzhou, China

**Keywords:** pepper, MAPK, MAPKK, genome-wide analysis, gene expression

## Abstract

The tripartite mitogen-activated protein kinase (MAPK) signaling cascades have been implicated in plant growth, development, and environment adaptation, but a comprehensive understanding of MAPK signaling at genome-wide level is limited in *Capsicum annuum*. Herein, genome-wide identification and transcriptional expression analysis of MAPK and MAPK kinase (MAPKK) were performed in pepper. A total of 19 pepper *MAPK* (*CaMAPKs*) genes and five *MAPKK* (*CaMAPKKs*) genes were identified. Phylogenetic analysis indicated that *CaMAPKs* and *CaMAPKKs* could be classified into four groups and each group contains similar exon-intron structures. However, significant divergences were also found. Notably, five members of the pepper MAPKK family were much less conserved than those found in Arabidopsis, and 9 Arabidopsis MAPKs did not have orthologs in pepper. Additionally, 7 MAPKs in Arabidopsis had either two or three orthologs in the pepper genome, and six pepper MAPKs and one MAPKK differing in sequence were found in three pepper varieties. Quantitative real-time RT-PCR analysis showed that the majority of *MAPK* and *MAPKK* genes were ubiquitously expressed and transcriptionally modified in pepper leaves after treatments with heat, salt, and *Ralstonia solanacearum* inoculation as well as exogenously applied salicylic acid, methyl jasmonate, ethephon, and abscisic acid. The MAPKK-MAPK interactome was tested by yeast two-hybrid assay, the results showed that one MAPKK might interact with multiple MAPKs, one MAPK might also interact with more than one MAPKKs, constituting MAPK signaling networks which may collaborate in transmitting upstream signals into appropriate downstream cellular responses and processes. These results will facilitate future functional characterization of MAPK cascades in pepper.

## Introduction

Plant growth, development, and response to environmental cues are tightly and coordinately regulated by sophisticated signaling networks. Protein phosphorylation mediated by protein kinase cascades is one of the most important post-translational modifications in networks that efficiently transduce input signals into suitable outputs. One of the most important protein-kinase-based amplification cascades is the mitogen-activated protein kinase (MAPK) cascade, typically comprising the hierarchically organized MAPK kinase kinase (MAPKKK)-MAPK kinase (MAPKK)-MAPK, all encoded by multiple genes (Smékalová et al., 2014). In this cascade, MAPKs phosphorylate specific substrate proteins such as transcription factors and enzymes and subsequently trigger cellular responses. MAPKs are activated when both tyrosine and threonine residues in the TEY/TDY motif are phosphorylated by dual-specificity kinases (MAPKKs), which in turn are activated by MAPKKKs via phosphorylation of the conserved Thr/Ser motif (Group, 2002; Ishihama et al., 2011; Janitza et al., 2012). These cascades of activations rapidly transform upstream signals into appropriate intracellular responses (Group, 2002). Given the presence of multiple genes encoding MAPKKK (MAPKKKs), MAPKK (MAPKKs), and MAPK (MAPKs) family members in genomes of different plant species, one MAPK cascade may be associated with more than one upstream or downstream partner (Cardinale et al., 2002). Indeed, a myriad of MAPK cascade formations are possible, which may enable these modules to regulate a variety of different physiological and developmental processes. The signals perceived by MAPKKKs are integrated and transduced by MAPKKs, and the specificities of cellular responses regulated by MAPK modules are mainly determined by MAPKs. Extensive studies have demonstrated that multiple abiotic stimuli, such as wounding and exposure to drought, cold, salinity (Sinha et al., 2011; Zhang et al., 2014), pathogens, and microbe-associated molecular patterns (MAMPs); (Pitzschke et al., 2009), affect the expression of MAPKs, MAPKKs, and MAPKKKs. However, few of these genes have been associated with specific physiological functions. Limited numbers of MAPK cascades have been functionally identified and characterized. Some examples include drought, cold, and salt stress signal-associated cascades, such as AtMEKK1-AtMKK1/AtMKK2-AtMPK4 (Ichimura et al., 2000; Desikan et al., 2001; Hadiarto et al., 2006; Kong et al., 2012b; Zhang et al., 2012; Furuya et al., 2014), MEKK1-MKK4/5-MPK3/6-WRKY22/WRKY29 (a module involved in plant innate immunity); (Asai et al., 2002), AtMKK2-AtMPK10 MAPK (a module that regulates venation complexity by altering polar auxin transport efficiency); (Stanko et al., 2014), and OsMKK4-OsMPK1-OsWRKY53 (a module involved in the wounding signaling pathway); (Yoo et al., 2014), have been reported. Some MAPKs such as OsMPK20-4 and OsMPK3 might interact with each other without phosphorylation (Sheikh et al., 2013), and some MAPK cascades might crosstalk with CDPK signaling (Mehlmer et al., 2010). In some case, cascades in MAPK modules such as MEKK1 can take a shortcut to directly interact with the transcription factor WRKY53 (Miao et al., 2007). These crosstalks and shortcuts complicate the MAPK modules, which may facilitate adaptation and survival of plants in the face of diverse environmental stresses. The precise regulation mechanisms mediated by MAPK modules remain to be elucidated.

The completion of the genome sequencing of different plant species has made it possible to identify and characterize all members of the MAPK, MAPKK, and MAPKKK families. 20 MAPKs in Arabidopsis (Group, 2002), 17 MAPKs in rice (Hamel et al., 2005; Rao et al., 2010), 19 MAPKs in maize (Kong et al., 2013a), and 10 MAPKs in mulberry (Wei et al., 2014) have been identified. Depending on phylogeny, MAPKs from different plant species can be classified into four subgroups (groups A, B, C, and D); (Janitza et al., 2012; Asif et al., 2014). The TEY MAPKs constitute groups A–C; whereas, the TDY MAPKs are included in group D, and the estimated number in group D is diverse among different species with 8 in Arabidopsis (Cardinale et al., 2002), 10 in rice (Hamel et al., 2006; Rao et al., 2010), 14 in soybean (Neupane et al., 2013b), 11 in maize (Kong et al., 2013a; Liu et al., 2013), 9 in poplar (Hamel et al., 2005), and 9 in *Brachypodium distachyon* (Chen et al., 2012). Members in group A, B, C, and D (Sinha et al., 2011; Šamajová et al., 2013) have been implicated in responses of plants to abiotic and biotic stresses. Phylogenetic analyses of the MAPKK gene families showed that all MAPKK genes formed four groups (groups A, B, C, and D). The amino acid sequence of MAPKKs phosphorylation sites in plants is different from that of mammals. Plant MAPKKs contain conserved S/TxxxxxS/T activation motif, whereas mammalian enzymes have S/TxxxS/T motif. MAPK modules appear to be conserved throughout the evolution of higher plants; indeed, MAPK, MAPKK, and MAPKKK in different plant species shared sequence similarity (Janitza et al., 2012), and these evolutionary relationships within subfamilies are supported by exon-intron organizations and the architecture of conserved protein motifs. Sequence diversity also exists between members of MAPK and MAPKK family members in the same genome, as well as across genomes of different plant species (Chen et al., 2012; Wu et al., 2014; Wang et al., 2015). Genome-wide analysis of the MAPKs and MAPKKs might provide insights into the evolutionary relationships within subfamilies of MAPK and MAPKK and their potential roles in regulating growth, development, and responses to biotic and abiotic stresses in different plant species.

Pepper (*Capsicum annuum*), a vegetable crop of great agricultural and economic importance, is a typical Solanaceae species and is susceptible to soil-borne diseases. Heavy losses in pepper production are frequently caused by various soil-borne diseases as well as different abiotic stresses, including drought, extreme temperatures and salinity. The benefit of investigating the molecular mechanism of disease or stress resistance/tolerance is genetic improvement of the crop to better resist these diseases and stresses. Functional characterization of MAPK modules is a feasible approach for dissecting the molecular mechanism of crop disease or stress resistance/tolerance. So far, only two MAPKs have been characterized in pepper. Shin et al. cloned 2 mitogen-activated protein kinase (CaMK1 and CaMK2, renamed as CaMPK3 and CaMPK6-1 in our study) from pepper leaves and showed that *CaMK1* and *CaMK2* can be induced by wounding, UV-C and cold treatments (Shin et al., 2001). Furthermore, Huh et al. found that CaMK1 and CaMK2 can both be induced by Tobacco mosaic virus (TMV) infection and interact with CaWRKYa and phosphorylate CaWRKYa and proved that CaWRKYa is regulated by CaMK1 and CaMK2 at the posttranslational level in hot pepper (Huh et al., 2015), indicating that MAPK cascades may play important roles in the response of pepper to biotic and abiotic stresses. To get a more comprehensive understanding of the roles of MAPK cascades in pepper growth, development, and environment adaption, a genome-wide identification and expression analysis of MAPKs and MAPKKs was performed in the present study by searching the newly published pepper genome sequences (Kim et al., 2014). A total of 19 MAPK genes and five MAPKK were identified, annotated, and named according to their sequence similarity with Arabidopsis genes, and their chromosomal position and gene structure were also determined. Additionally, their transcript profiles in different organs were analyzed using published RNA-seq data, and their expression patterns in response to salt stress, heat shock, and *Ralstonia solanacearum* (RS) inoculation and exogenous application of phytohormones were also investigated using quantitative real time polymerase chain reaction (qPCR).

Our data showed that despite comparable structural conservation displayed by the conserved domains typically found in MAPKs and MAPKKs from other plant species, different degree of sequence divergences were also found in different MAPKs and MAPKKs between pepper and other plant species. Additionally, different transcriptional expressions of the members of MAPK and MAPKK families were found in different pepper organs and in the responses of pepper to the tested biotic and abiotic stresses, suggesting their important roles in the regulation of pepper growth, development, and responses to the tested biotic or abiotic stresses. To our knowledge, this is the first reported genome-wide and expression analysis of the pepper MAPK and MAPKK families, which would provide valuable information for further functional investigations of pepper MAPK and MAPKK families.

## Materials and methods

### Identification of MAPK and MAPKK in pepper

The completed genome sequences, CDS sequences, and peptide sequences of pepper version 1.55 were downloaded from the website (http://peppergenome.snu.ac.kr/) to construct a local genome database. To find all MAPK and MAPKK family members in pepper, NCBI BLASTP searches against a local database built using protein sequences were performed using sequences from all 35 known MAPKKs from Arabidopsis, tomato, rice, and soybean and 90 MAPKs from the above mentioned plant species (Table S1). In addition, a hidden Markov model (HMM) profile of these MAPK and MAPKK was generated using the Hmmer v 3.2 program (Finn et al., 2011) and was used as database to search similar proteins in the pepper protein database, sequences got from BLASTP search and analysis were further verified by HMM. Only the proteins with greater than 50% sequence identity were collected. Redundant sequences were removed manually, and the candidate sequences were further evaluated by identifying the putative functional domains using NCBI BLASTP (http://blast.ncbi.nlm.nih.gov/) and Arabidopsis information resource (http://www.arabidopsis.org/) and the domain analysis software SMART (Simple Modular Architecture Research Tool: http://smart.emblheidelberg.de/). Sequences without relevant domains or conserved motifs were removed. After multiple cycles of these analyses, MAPKK and MAPK family members in pepper were identified. All collected proteins were subjected to protein blast in the Arabidopsis information resource (http://arabidopsis.org/) for their nomenclature. The parameters of the deduced MAPK and MAPKK proteins, such as isoelectric points and molecular weights, were calculated on the ExPASy Proteomics Server (http://web.expasy.org/compute_pi/).

### Analyses of chromosomal locations, gene structures, and promoter regions

Chromosomal locations and gene structures were obtained and analyzed from genome data downloaded from the pepper genome database (http://peppergenome.snu.ac.kr/ and http://peppersequence.genomics.cn/page/species/index.jsp). The promoter regions of the MAPK and MAPKK family members were identified from the whole-genome shotgun configurations available from the NCBI database. *Cis-element* analysis of the MAPK and MAPKK promoters were completed using the PlantCARE software (http://bioinformatics.psb.ugent.be/webtools/plantcare/html/). The number of responsive *cis*-elements was calculated.

### Sequence homolog alignment and phylogenetic analysis

To examine the evolutionary relationships between different MAPK and MAPKK members in pepper and Arabidopsis, the protein sequences of Arabidopsis and pepper MAPKs and MAPKKs were collected from the Arabidopsis information resource and pepper genome database (http://peppergenome.snu.ac.kr/), respectively. Phylogenetic analysis was performed using the Maximum likelihood method with bootstrapping analysis in the software MEGA version 6.0 (Kumar et al., 2008) and the bootstrap test was carried out with 1000 replicates.

### Orthologs sequence analysis of MAPKS and MAPKKS among pepper, tomato, arabidopsis, and rice

To investigate the evolutionary relationships of MAPKs and MAPKKs among pepper, tomato, Arabidopsis and rice, the protein sequences of MAPKs and MAPKKs in pepper and their orthologs with highest sequence identities in tomato, Arabidopsis and rice were collected from the genome databases of pepper (http://peppergenome.snu.ac.kr/), tomato (http://solgenomics.net/tools/blast/), Arabidopsis (http://www.arabidopsis.org/), and rice (http://rice.plantbiology.msu.edu/analyses_search_blast.shtml), respectively. And the software DNAMAN was used to make multiple sequence alignment on MAPKs and MAPKKs of pepper with their orthologs in other plant species.

### Plant materials and growth conditions

The local cultivation pepper line 68-2 (from Quanzhou, Fujian, China) was used as our plant material. The pepper seeds were sowed on the soil for germination. After germination, the seedlings were transferred to the greenhouse and grown under standard conditions (16 h of light and 8 h of dark at 25°C), and 4-leaf-old pepper plants were used for treatment. For high-temperature treatment, the pepper plants were grown in a climate chamber at either high temperature (42°C) or normal temperature (25°C) for 24 h. For RS pathogen treatment, the plants were inoculated with a suspension of RS (cfu = 10^8^) and maintained in a climate chamber at 28°C for 24 h. For plant hormones (ABA, SA, MeJA, and ETH) treatment, pepper plants were sprayed with either 100 μM abscisic acids (ABA), 1 mM salicylic acid (SA), 100 μM methyl jasmonate (MeJA), or 100 μM ethephon (ETH) dissolved in ddH_2_O. Meanwhile, control plants were sprayed with ddH_2_O. All plants were kept at 25°C for 24 h. Three repetitions were performed for each of the above groups. After treatment, the leaves were collected for RNA extraction. The pepper plant RNA-seq data of the two gene families (obtained from different tissues of pepper plants) were obtained from a previously published paper (Kim et al., 2014).

### Quantitative real-time PCR analysis

The RNA-seq data of gene expression in different tissues in CM334 pepper plants was downloaded from Supplementary Table 22 of Kim's paper (Kim et al., 2014). Three individual plants were harvested for RNA extraction per treatment. The harvested leaves were frozen immediately by liquid nitrogen and transferred to the –80°C freezer. Frozen leaf tissues were disrupted in 2 mL RNAse-free microcentrifuge tubes (Axygen, USA) using three stainless steel beads and the Tissue Lyser II (Qiagen, Dusseldorf, Germany). Trizol (Invitrogen, Carlsbad, USA) was used to extract the total RNA from the disrupted tissues. Both RNA concentration and quality were confirmed by the NanoDrop 2000 (ThermoScientific, Massachusetts, USA). The RNA integrity was confirmed by agrarose electrophoresis. RNA (500 ng from each sample), 250 ng of oligo dT(15) primer, and 200 unit reverse transcriptase was used for a reverse transcription reaction using One Step PrimeScript™ cDNA Synthesis Kit (TaKaRa, Shigo, Japan), which include a genomic DNA digestion step, with the following programme: 42°C, 60 min; 85°C, 5 s; 4°C, forever. The synthesized cDNA products were diluted ten-fold for further qPCR analysis. qPCR was performed as a 20-μL reaction (10 μL 2 × SYBR Premix Ex Taq, 0.2 μM forward and reverse primers, and 100 ng cDNA) in a 200-μL PCR tube (Axygen, Hangzhou, China) using SYBR Premix Ex Taq™ (TaKaRa) and a CFX™ Real-Time System (Bio-rad, Hercules, USA) with the following program: 95°C, 30 s; 95°C, 5 s; 60°C, 34 s; 95°C, 15 s, 40 cycles. SYBR green fluorescence was used to detect the amplification of the target genes at each cycle. The Cq (threshold cycle), defined as the real-time PCR cycle at which a statistically significant increase of reporter fluorescence was first detected, was used as a measure for the starting copy numbers of the target gene. Three replicates of each experiment were performed, and three replicates were performed using independent plant individuals. Data were analyzed by the Livak method (Livak and Schmittgen, 2001; Bustin et al., 2009), and expressed as a normalized relative expression level (2^−ΔΔCq^) of the respective genes. *CaActin* (GQ339766) and 18S ribosomal RNA (EF564281), which have been confirmed by the published paper (Choi et al., 2012; Kim et al., 2015) and further confirmed by our own experiment, were used to normalize the transcript levels in pepper. Student's *t*-test was used to determine the statistical significance of the differential expression patterns between treatments and controls. Gene specific primers (GSPs) used in qPCR analyses are listed in Table S2 and the quality and specificity of the primers were confirmed by the software primer primer 5.0 and the genome database of pepper plants (http://peppergenome.snu.ac.kr/); (Table S1), respectively. The specificity of the PCR products was confirmed by the melting curve and the qPCR validation was also performed using the Bio-rad CFX manager 2.0 (Table S2).

### Yeast two-hybrid assays

The interactions between MAPKs and MAPKKs proteins were identified in the Gal-4 reporter-based ProQuest™ two-hybrid system (Invitrogen, Darmstadt, Germany). CDS coding for all the MAPKs members of pepper were cloned into the prey vector (pDEST32) and CDS coding for MAPKKs were cloned into the bait vector (pDEST22). All the combinations of each bait and prey plasmid were PEG-transformed into the yeast strain MaV203 following the protocol of ProQuest™ two-hybrid system. Positive transformants were first selected in the synthetic dropout (SD) medium without leucine and tryptophan (SD/-Leu/-Trp) and the culture was transferred to the selection medium without Leucine, tryptophan, histidine and adenine. 3-amino-1,2,4-triazole (3-AT) was added to the selection plates to suppress the auto-activation of the prey vectors.

## Results

### Identification of MAPK and MAPKK genes in the pepper genome

The release of the complete pepper genome sequences makes it possible to identify pepper MAPK and MAPKK genes in a genome-wide manner (Kim et al., 2014). In the present study, the deduced amino acid sequences of MAPKs and MAPKKs from Arabidopsis, rice, tomato and soybean were used as queries to search for orthologs by BLASTP in the genome of pepper cultivar CM334, the redundant candidate sequences or sequences without relevant domains or conserved motifs were deleted manually. Sequences got from BLASTP search and analysis were further verified by HMM result. Finally, 19 *CaMAPKs* and five *CaMAPKKs* were acquired using this method. The protein sequences of the CaMAPKs and CaMAPKKs were further characterized by conserved domain analysis using the SMART software. The newly identified MAPK and MAPKK genes were designated following the nomenclature of *Arabidopsis thaliana* according to sequence similarities (Table 1). The number of MAPKs in pepper was similar to that of tomato (Kong et al., 2012a), *Brachypodium distachyon* (Chen et al., 2012), rice (Hamel et al., 2006; Rao et al., 2010), Arabidopsis (Asai et al., 2002; Colcombet and Hirt, 2008; Popescu et al., 2009), banana (Asif et al., 2014), and apple (Zhang et al., 2013); (Table 2). In contrast, the five MAPKKs in pepper were the same amount found in tomato (Wu et al., 2014), but differed significantly in number to the MAPKKs found in Arabidopsis, rice (Hamel et al., 2006; Rao et al., 2010), *Brachypodium distachyon* (Chen et al., 2012), and apple (Zhang et al., 2013). Sequence analysis revealed that several MAPKs in Arabidopsis, such as *AtMPK2, 5, 8, 10, 11, 12, 14, 17, 18*, had no orthologs in pepper; *AtMPK4, 6, 9, 13, 15, 19*, and *20* had more than one ortholog in the pepper genome. These orthologs in pepper were designated as *CaMPK4-1, 4-2, 4-3, 6-1, 6-2, 9-1, 9-2, 13-1, 13-2, 17-1, 17-2, 19-1, 19-2, 20-1*, and *20-2* based on their sequence similarities. Similarly, five MAPKKs had orthologs in the pepper genome, and these were designated as *CaMKK2*, –*3*, –*5*, –*6* and –*9* based on sequence similarities to their orthologs in Arabidopsis. The detailed information of two gene family members, including the number of introns, genome ID, genomic position, number of deduced amino acid residues, number of nucleotides, molecular weight, and isoelectric point (pI), is shown in Table 1. ORF (open reading frame) lengths of MAPK and MAPKK genes ranged from 984 to 1935 bp and 984 to 1140 bp, respectively, and the molecular weight of the resulting proteins ranged from 26.14 to 70.71 kDa and 36.76 to 41.72 kDa, respectively. The pIs of MAPKs and MAPKKs ranged from 5.06 to 9.54 and 5.68 to 8.89, respectively. The protein lengths of identified MAPKs in apple and maize were 370 amino acids (aa) to 865 aa and 369 aa to 615 aa, respectively and the corresponding pIs were 5.07–9.54 and 5.26–9.72 (Liu et al., 2013; Zhang et al., 2013). The lengths of identified MAPKKs in apple and maize were 314–554 aa and 227–613 aa and their pIs were 5.27–9.2 and 5.26–9.72, respectively (Liu et al., 2013; Zhang et al., 2013).

**Table 1:** **Characteristics and nomenclature of pepper MAPK and MAPKK**.

**Family family**	**Gene**	**Introns**	**Genome ID v1.55**	**I homologous gene**	**Homologous Gene ID**	**Chr**	**Genomic position**	**ORF (bp)**	**size(aa)**	**MW (Da)**	**pi**
MAPK	CaMPK1	1	CA04g21490	AtMPK1	AT1G10210	4	218599170–218603799	1119	372	42755.41	6.32
	CaMPK3	5	CA06g06270	AtMPK3	AT3G45640	6	59521988–59525433	1128	375	42926.19	5.40
	CaMPK4-1	5	CA08g07540	AtMPK4	AT4G01370	8	124018835–124024649	1122	373	42918.80	5.97
	CaMPK4-2	1	CA01g02140	AtMPK4	AT4G01370	1	3173279–3177283	1140	379	43679.94	6.18
	CaMPK4-3	5	CA05g12170	AtMPK4	AT4G01370	5	169095789–169099946	1131	376	43070.13	6.97
	CaMPK6-1	5	CA08g04480	AtMPK6	AT2G43790	8	82185530–82158832	1179	393	45042.52	5.48
	CaMPK6-2	4	CA03g14570	AtMPK6	AT2G43790	3	158852213–158856921	1221	406	46279.42	6.70
	CaMPK7	1	CA02g22080	AtMPK7	AT2G18170	2	156967714–156970175	1113	370	42743.57	8.29
	CaMPK9-1	6	CA12g10140	AtMPK9	AT3G18040	12	78469839–78474912	1698	566	35048.30	8.78
	CaMPK9-2	0	CA06g17760	AtMPK9	AT3G18040	6	216070086–216075975	1653	550	42355.71	8.08
	CaMPK13-1	4	CA11g13970	AtMPK13	AT1G07880	11	228132171–228137306	990	329	39198.82	9.54
	CaMPK13-2	5	CA11g13960	AtMPK13	AT1G07880	11	228131951–228132133	1119	372	42763.81	5.06
	CaMPK16	6	CA01g24290	AtMPK16	AT5G19010	1	192066037–192070249	1260	419	48384.79	8.37
	CaMPK17-1	8	CA05g08630	AtMPK15	AT1G73670	5	67468808–67475261	1482	493	56304.81	7.00
	CaMPK17-2	7	CA00g57490	AtMPK15	AT1G73670	11	227489423–227492938	1704	568	64746.09	7.21
	CaMPK19-1	8	CA07g16590	AtMPK19	AT3G14720	7	221712314–221715958	1884	627	70709.40	9.25
	CaMPK19-2	8	CA10g01850	AtMPK19	AT3G14720	10	3904073–3908183	1809	603	68242.22	9.09
	CaMPK20-1	8	CA07g15380	AtMPK20	AT2G42880	7	217189491–217192973	1860	619	70355.78	9.14
	CaMPK20-2	1	CA07g14780	AtMPK20	AT2G42880	7	206746721–206747523	1935	644	26143.11	8.66
MAPKK	CaMKK2	7	CA00g74230	AtMKK2	AT4G29810	9	5779660–5782151	1074	357	39435.06	5.68
	CaMKK3	5	CA03g23860	AtMKK3	AT5G56580	3	227498017–227503127	1548	515	57392.65	5.63
	CaMKK5	0	CA03g36820	AtMKK5	AT3G21220	3	257411669–257412808	1140	379	41717.43	8.86
	CaMKK6	7	CA00g86340	AtMKK6	AT5G56580	3	10208073–10210986	1116	371	41585.83	5.89
	CaMKK9	0	CA03g22790	AtMKK9	AT1G73500	3	224,956,092–224957075	984	327	36757.45	8.89

**Table 2:** **The distribution of MPKs in Arabidopsis, Populus, rice, pepper, Brachypodium, and apple**.

	***Arabidopsis thaliana***	***Populus trichocarpa***	***Oryza sativa***	***Capsicum annuum***	***Brachypodium distachyon***	***Malus domestica***
Group A	3	4	2	3	2	5
Group B	5	4	1	5	2	6
Group C	4	4	2	2	3	5
Group D	8	9	10	9	9	10

### Multiple alignments, phylogenetic analysis, and domain analysis of MAPK and MAPKK families in the pepper genome

**Figure 1:**
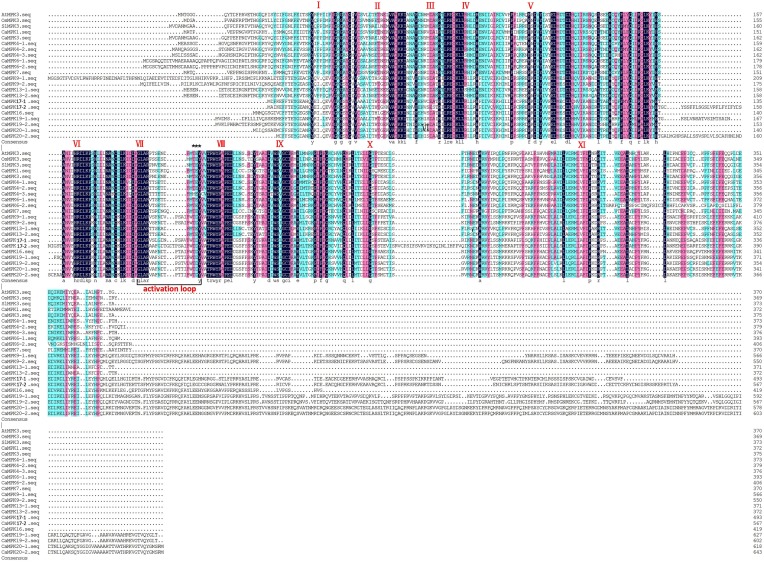
**Multiple sequence alignment of the amino acid sequences of pepper MAPKs and MAPKs from other plant species (tomato, Arabidopsis, and rice)**. The alignment was performed using Clustal X. The 11 conserved domains (I–XI) present in pepper serine/threonine protein kinases are denoted by Roman numerals. The activation loop region is marked by a black line. The conserved threonine and tyrosine residues are indicated by asterisks.

**Figure 2:**
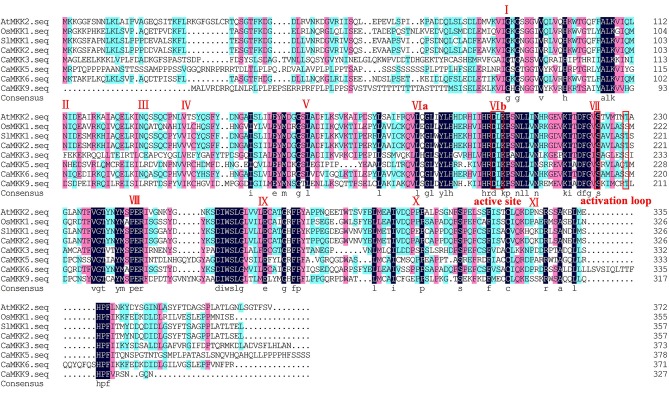
**Alignment of multiple pepper MAPKK amino acid sequences from pepper and other plant species (tomato, Arabidopsis, and rice)**. The alignment was performed using Clustal X. The 11 conserved domains (I–XI) present in pepper serine/threonine protein kinases are denoted by Roman numerals. The conserved S/T-X5-S/T motif and active site D (I/L/V) K motif are highlighted by a red box.

The alignments of the 19 MAPKs and five MAPKKs of pepper were performed using clustal X. The results of the alignment indicated that all 19 identified MAPKs contain 11 domains (I-XI) that are conserved in the serine/threonine protein kinases from other plant species (Figure 1). Several threonine and tyrosine residues located between domains VII and VIII of all 19 pepper MAPKs were conserved when compared to those of other plant species. These constitute the activation loop and are known to be phosphorylated during MAPK activation. Of the 19 MAPKs, nine contained the TDY motif, another nine contained the TEY motif, and only CaMPK4-3 contained the MEY motif. These results are consistent with that in rice and tomato, which each contains only one MAPK with a MEY domain (Zhang et al., 2013). Additionally, the majority of the TDY MAPKs had long C-terminal extensions compared to the TDY MAPKs in the pepper genome, which was consistent with the MAPKs from the other plant species (Zhang et al., 2013). The alignment of the five pepper MAPKKs showed the existence of 11 conserved motifs including S/T-X5-S/T (activation loop) and the DIK motif (active site); (Figure 2). These motifs were previously found in MAPKKs from other plant species (Hamel et al., 2006).

**Figure 3:**
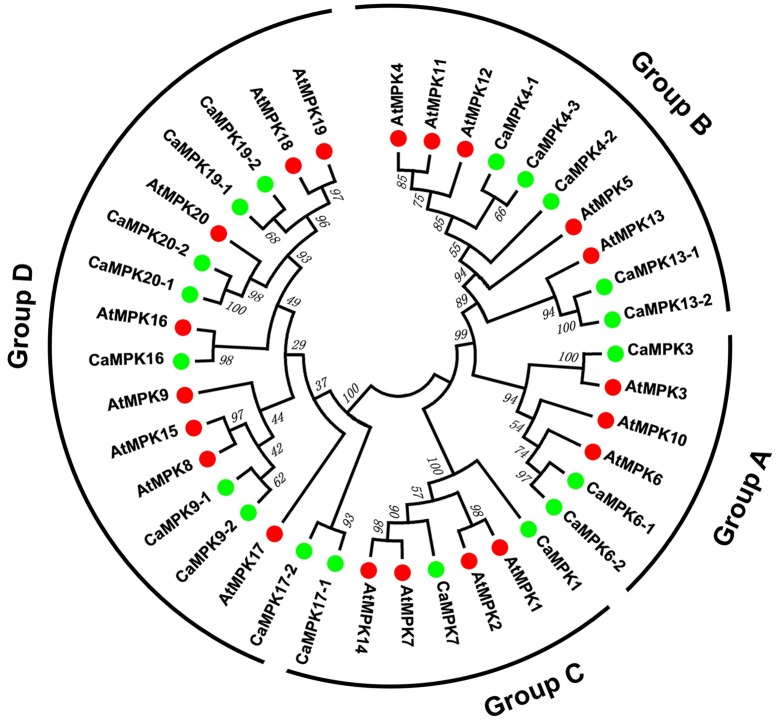
**Phylogenetic relationship among MAPK genes from pepper and Arabidopsis**. The complete amino acid sequences of 19 pepper MAPKs and 20 Arabidopsis MAPKs were aligned using Clustal X, and the Maximum Likelihood tree was constructed using MEGA 6.0 with bootstrapping analysis (1000 replicates). The values above the branches are bootstrap support of 1000 replicates. Groups A–D indicate different gene clusters. To identify the plant species origin for each MAPK, a species acronym is included before the protein name: CaMAPK indicate MAPK from pepper and AtMAPK indicate MAPK from Arabidopsis. The green and red dots before the protein names indicate MAPKs from pepper and Arabidopsis, respectively.

**Figure 4:**
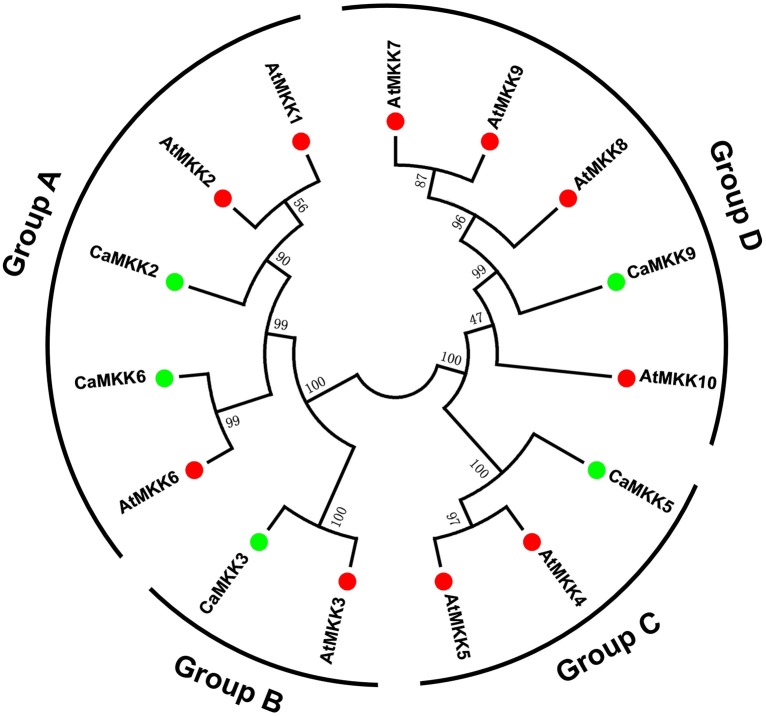
**Phylogenetic relationship among MAPKK genes from pepper and Arabidopsis**. The complete amino acid sequences of five pepper MAPKKs combined with 10 Arabidopsis MAPKKs were aligned by Clustal X, and the Maximum Likelihood tree was constructed using MEGA 6.0 with bootstrapping analysis (1000 replicates). The values above the branches are bootstrap support of 1000 replicates. Groups A–D indicate different gene clusters. To identify the plant species origin for each MAPKK, a species acronym is included before the protein name: CaMKK indicate MAPKK from pepper and AtMKK indicate MAPKK from Arabidopsis. The green and red dots before the protein names indicate MAPKKs from pepper and Arabidopsis, respectively.

To analyze the evolutionary relationships between different MAPK and MAPKK family members in pepper and Arabidopsis, full-length amino acid sequences of 19 MAPKs and five MAPKKs from pepper, and 20 MAPKs and 10 MAPKKs from Arabidopsis, were together subjected to a multiple sequence alignment using the MEGA 6.0 program. An unrooted phylogenetic tree was constructed, and the 19 MAPKs were divided into four groups (A, B, C, and D) based on their predicted amino acid sequences (Figure 3). The size of groups A and B appeared to be similar; whereas, the size of group D differed significantly between pepper and Arabidopsis. The group D gene family is the largest subfamily in rice, poplar, tomato, and apple (Chen et al., 2012; Zhang et al., 2013; Wang et al., 2015). The five MAPKKs in the pepper genome were also classified into four groups with the MAPKKs of Arabidopsis. Like the MAPKKs in tomato (Wu et al., 2014), two MAPKKs were classified into group A, and groups B, C, and D each had one MAPKK in pepper (Figure 4). Similar to MAPKs, the number of MAPKKs in group D differed greatly between pepper and Arabidopsis.

### Genomic distribution and organization of MAPK and MAPKK family genes

**Figure 5:**
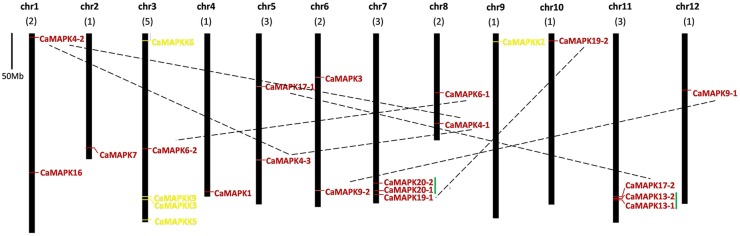
**Positions of MAPK and MAPKK gene family members on pepper chromosomes**. The chromosome number is indicated at the top of each chromosome representation. Paralogs are indicated by green line. Scale represents a 50 Mb chromosomal distance. The paired MAPK genes are marked with a broken line.

The chromosomal locations of pepper MAPK and MAPKK genes were determined using BLASTN analysis of pepper chromosomes. The 19 *CaMAPK* genes were dispersed throughout 11 of the 12 chromosomes (Figure 5). Chromosomes 7 and 11 each contain 3 MAPK family genes, while chromosomes 1, 5, 6, and 8 each contain two MAPKs. Chromosomes 2, 3, 4, 10, and 12 each contain one MAPK. Among these MAPKs, two pairs of paralogous genes (*CaMPK13-1*/*CaMPK13-2* and *CaMPK20-1*/*CaMPK20-2*) were located together on chromosomes 11 and 7, respectively. Other paralogs (*CaMPK4-1*/*CaMPK4-2/CaMPK4-3, CaMPK6-1/CaMPK6-2, CaMPK17-1/CaMPK17-2*, and *CaMPK19-1/CaMPK19-2*) were located on different chromosomes. Four of the five MAPKK family members, *CaMKK3, CaMKK5, CaMKK6*, and *CaMKK9* were located on chromosome 3, while *CaMKK2* was located on chromosome 2 (Figure 5). A similar observation was reported for tomato, where four MAPKKs were present on one chromosome and another MAPKK was located on a different chromosome (Wu et al., 2014).

**Figure 6:**
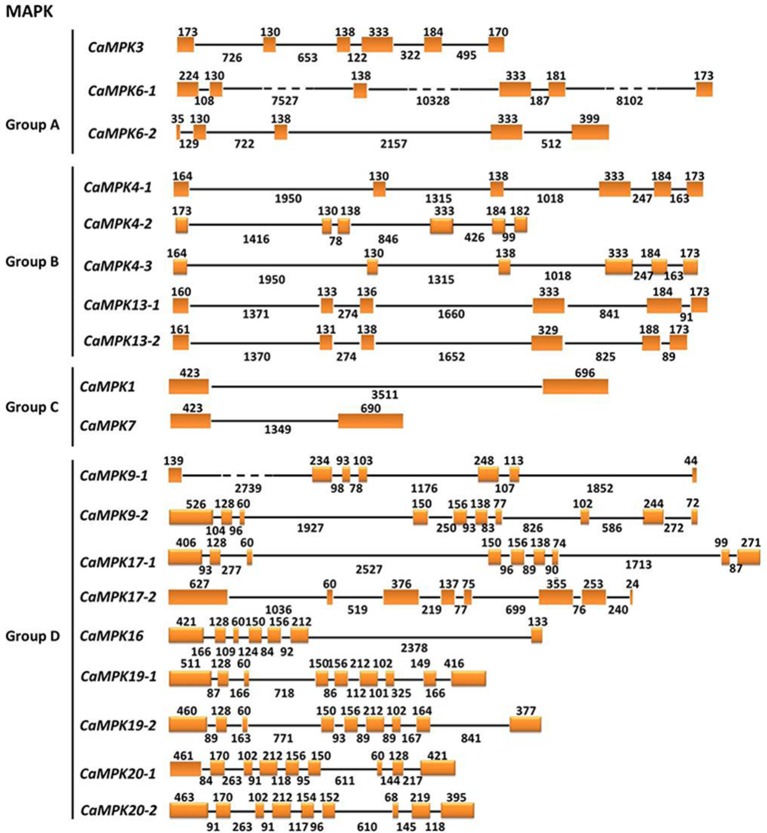
**The exon/intron structure of pepper MAPK genes**. Introns and exons are represented by black lines and orange boxes, respectively. Introns and exons of the CaMAPKs are grouped according to the phylogenetic classification. The length in base pairs of each intron and exon is also indicated.

**Figure 7:**
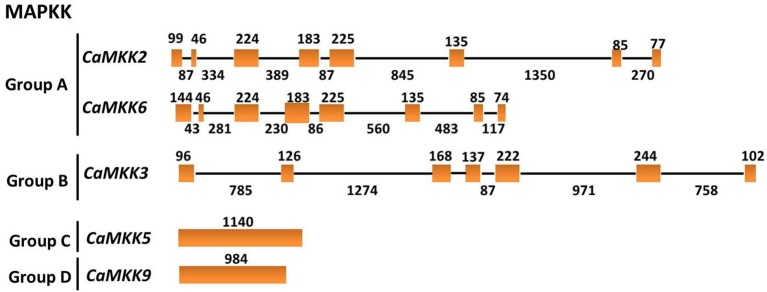
**The exon/intron structure of pepper MAPKK genes**. Introns and exons are represented by black lines and orange boxes, respectively. Introns and exons of the CaMAPKKs are grouped according to the phylogenetic classification. The length in base pairs of each intron and exon is also indicated.

To get insights into the gene structures of the MAPK and MAPKK gene families, the exon/intron organization of the two gene families was analyzed. MAPKs within the same groups exhibit similar exon-intron organizations. For example, MAPKs in group A contain 5–6 exons and 4–5 introns; MAPKs in group B contain 6 exons and 5 introns. MAPKs in group C contain 2 exons and one intron, and MAPKs in group D contain 7–10 exons and 6–9 introns (Figure 6). Similar to tomato, the MAPKKs in group A contain 8 exons and 7 introns. The group B MAPKK contains 6 exons and 5 introns, but MAPKK genes from groups C and D contain no introns (Figure 7).

### Sequence similarities of MAPKS and MAPKKS among pepper, tomato, arabidopsis, and rice

To investigate the evolutionary relationships of MAPKs and MAPKKs in pepper with their orthologs in other plant species, the sequences of MAPKs and MAPKKs in pepper genome were compared to their corresponding orthologs in tomato, Arabidopsis and rice (Table 3). Differences in the sequence similarities were found among different MAPK and MAPKK family members, for example, CaMPK4-3 and CaMKK9 both share more than 98.00% sequence identities with their orthologs in tomato, whereas, CaMPK16 and CaMKK3 both share only around 70% sequence identities with their orthologs in tomato. CaMPK7 and CaMKK6 share more than 86 and 73% sequence identities with their orthologs in rice, whereas, CaMPK17-2 and CaMKK3 share only around 50 and 16% with their orthologs in rice, respectively, suggesting that members in MAPK and MAPKK families in pepper exhibit different evolution rates. In addition, the sequence identities of orthologs in MAPK and MAPKK family between pepper and tomato are generally higher than those between pepper and Arabidopsis, and the sequence identities of orthologs of MAPK and MAPKK families between pepper and Arabidopsis are higher than those between pepper and rice. For example, the sequence identities of orthologs in MAPKs and MAPKKs between pepper and tomato range from 71.68 to 99.20% with an average of 89.70 and 69.32 to 98.25% with an average of 88.94%, respectively. The sequence identities of orthologs in MAPK and MAPKK families between pepper and Arabidopsis range from 54.56 to 87.12% (with an average of 73.57%) and 57.50 to 80.16% (with an average of 66.97%), respectively. While the sequence identities of orthologs in MAPK and MAPKK families between pepper and rice range from 50.00 to 78.17% (with an average of 68.81%) and 16.48 to 73.99% (with an average of 51.32%). But the sequence similarities of MAPK and MAPKK genes in pepper to their orthologs in Arabidopsis were not always higher than their sequence similarities to the corresponding orthologs in rice. For example, the sequence similarity of CaMPK1 and AtMPK1 is lower than that of CaMPK1 and OsMPK7. These results suggest that sequence similarities of orthologs in MAPK and MAPKK families in different plant species are dependent on the period of time that they have diverged.

**Table 3:** **Sequences similarities of MPKs and MKKs among pepper, tomato, Arabidopsis, and rice**.

**Pepper**		**Rice**			**Tomato**			**Arabidopsis**		
**Gene name**	**Genome ID v1.5**	**Gene name**	**Os gene ID**	**Homology (%)**	**Gene name**	**Sl gene ID**	**Homology (%)**	**Gene name**	**AT gene ID**	**Homology (%)**
CaMPK1	CA04g21490	OsMPK7	Os06g48590	86.03	SlMPK9	Solyc04g080730	97.58	AtMPK1	AT1g10210	83.37
CaMPK3	CA06g06270	OsMPK6	Os06g06090	67.92	SlMPK3	Solyc06g005170	95.20	AtMPK3	AT3g45640	79.47
CaMPK4-1	CA08g07540	OsMPK4	Os10g38950	78.17	SlMPK5	Solyc01g094960	95.98	AtMPK4	AT4g01370	86.17
CaMPK4-2	CA01g02140	OsMPK4	Os10g38950	73.80	SlMPK7	Solyc08g081490	93.67	AtMPK4	AT4g01370	79.16
CaMPK4-3	CA05g12170	OsMPK4	Os10g38950	75.13	SlMPK6	Solyc05g049970	99.20	AtMPK4	AT4g01370	81.12
CaMPK6-1	CA08g04480	OsMPK6	Os06g06090	85.80	SlMPK2	Solyc08g014420	96.95	AtMPK6	AT2g43790	87.12
CaMPK6-2	CA03g14570	OsMPK6	Os06g06090	72.22	SlMPK2	Solyc08g014420	80.39	AtMPK6	AT2g43790	75.61
CaMPK7	CA02g22080	OsMPK6	Os06g06090	48.77	SlMPK7	Solyc02g084870	96.76	AtMPK7	AT2g18170	79.73
CaMPK9-1	CA12g10140	OsMPK17-1	Os06g49430	71.21	SlMPK9	Solyc12g040680	90.78	AtMPK9	AT3g18040	70.98
CaMPK9-2	CA06g17760	OsMPK17-1	Os06g49430	68.95	SlMPK9	Solyc06g068990	81.73	AtMPK9	AT3g18040	76.91
CaMPK13-1	CA11g13970	OsMPK4	Os10g38950	63.83	SlMPK4	Solyc11g072630	81.45	AtMPK13	AT1g07880	66.12
CaMPK13-2	CA11g13960	OsMPK4	Os08g06060	68.78	SlMPK4	Solyc11g072630	95.70	AtMPK13	AT1g07880	72.85
CaMPK16	CA01g24290	OsMPK16	Os11g17080	72.13	SlMPK13	Solyc01g080240	71.68	AtMPK16	AT5g19010	65.78
CaMPK17-1	CA05g08630	OsMPK16	Os11g17080	64.14	SlMPK14	Solyc04g007710	91.21	AtMPK17	AT2g01450	66.27
CaMPK17-2	CA00g57490	OsMPK21-1	Os05g50120	50.00	SlMPK15	Solyc05g008020	75.87	AtMPK17	AT2g01450	54.56
CaMPK19-1	CA07g16590	OsMPK20-4	Os01g47530	58.90	SlMPK11	Solyc07g062080	88.39	AtMPK19	AT3g14720	66.56
CaMPK19-2	CA10g01850	OsMPK20-4	Os01g47530	60.89	SlMPK10	Solyc10g007500	90.70	AtMPK19	AT3g14720	65.96
CaMPK20-1	CA07g15380	OsMPK20-1	Os01g43910	72.48	SlMPK12	Solyc07g056350	93.43	AtMPK20	AT2g42880	71.88
CaMPK20-2	CA07g14780	OsMPK20-1	Os01g43910	68.24	SlMPK12	Solyc07g056350	88.13	AtMPK20	AT2g42880	68.13
CaMKK2	CA00g74230	OsMKK1	Os06g05520	62.91	SlMPKK1	Solyc12g009020	94.96	AtMKK2	AT4g29810	67.20
CaMKK3	CA03g23860	OsMKK3	Os06g27890	49.14	SlMPKK5	Solyc03g019850	69.32	AtMKK3	AT5g56580	57.50
CaMKK5	CA03g36820	OsMKK4	Os02g54600	58.54	SlMPKK2	Solyc03g123800	88.39	AtMKK5	AT3g21220	67.80
CaMKK6	CA00g86340	OsMKK6	Os01g32660	73.99	SlMPKK3	Solyc03g119490	93.80	AtMKK6	AT5g56580	80.16
CaMKK9	CA03g22790	OsMKK5	Os06g09180	44.67	SlMPKK4	Solyc03g097920	98.25	AtMKK9	AT1g73500	62.20

### Sequence differences of MAPKS and MAPKKS among different pepper cultivars

To study the evolution speed of MAPKs and MAPKKs in pepper genomes, the sequences of MAPKs and MAPKKs of CM334 released by Kim et al. (2014) were compared to those in two other cultivars, *Chiltepin* (*Capsicum annuum* var. *glabriusculum*) and *Zunla-1* (*Capsicum annuum* L.), released by Qin et al. (2014); (Figure S1), as well as compared to the corresponding orthologs in Arabidopsis and tomato. The deduced amino acid sequences of the six MAPK genes, including CaMPK4-3, 17-1, 17-2, 19-1, 19-2, and 20-1 showed differences among the three pepper cultivars. For example, a fragment in the CDS of *CaMPK4-3* encoding 20 amino acid residues was lost in CM334. In contrast, two fragments encoding 17 and 21 amino acid residues in the CDS of *CaMPK19-1* were present in CM334 but were lost in *Chiltepin* and *Zunla-1*. Within the five MAPKKs, only CaMKK6 show differences among the three pepper cultivars.

### *Cis*-Element analysis in the promoter regions of MAPK and MAPKK genes in pepper

To investigate the possible roles of MAPKs and MAPKKs identified in the pepper genome, corresponding promoter regions (approximately 2 kb in length, the first nucleotide of the initiating ATG is designated +1) of the 19 MAPK and five MAPKK genes were subjected to *cis*-element analysis by PlantCARE online (Table 4). *Cis*-elements responsive to biotic [including W-box (Eulgem et al., 1999), EIRE (Fukuda, 1997), S box (pathogen-related *cis*-element Kunitomo et al., 2000)], and abiotic stresses [HSE (Mosser et al., 1988) and MBS (Nakagoshi et al., 1989)], as well as phytohormones such as SA [salicylic acid, TCA-element (Goldsbrough et al., 1993), JA jasmonic acid, CGTCA-motif (Beaudoin and Rothstein, 1997)], ET [ethylene, ERE (Itzhaki et al., 1994)], and ABA [abscisic acid, ABRE (Hamel et al., 2006)], were widely present in the promoters of the two gene families (Table 4). Several other *cis*-elements (listed in Table 4) were also found to be present only in the promoters of either MAPK genes or MAPKK genes, suggesting that transcriptional expression of MAPKK and MAPK genes may sometimes be co-regulated, but, at other times, regulated specifically.

**Table 4:** **Analysis of c/s -elements in the upstream promoter of MPK and MKK family genes in pepper**.

**Gene family**	**Gene name**	**ABRE**	**ARE**	**AuxRR-core**	**CGTCA-motif**	**DRE**	**EIRE**	**ERE**	**GARE-motif**	**GC-motif**	**GCC box**	**HSE**	**LTR**	**MBS**	**box**	**SP-box**	**TATC-box**	**TC-rich repeats**	**TCA-element**	**TGA-element**	**W box**	**WUN-motif**	**Total**
MAPKs	CaMPK1	1	2	0	1	0	0	1	0	0	0	1	2	0	0	0	0	0	0	0	4	0	12
	CaMPK3	1	1	0	2	0	0	0	0	0	1	1	0	2	0	0	0	0	3	0	1	0	12
	CaMPK4-1	0	3	0	0	0	0	0	0	1	0	2	0	0	0	0	0	2	0	1	0	0	9
	CaMPK4-2	1	0	0	1	0	0	0	0	0	0	4	0	1	0	0	0	2	1	0	1	0	11
	CaMPK4-3	0	5	2	0	0	1	1	0	0	0	1	0	1	0	1	0	1	0	3	0	0	16
	CaMPK6-1	0	0	0	1	0	0	2	0	0	0	0	1	1	0	0	0	3	1	0	2	0	11
	CaMPK6-2	1	1	0	3	0	0	0	2	0	0	0	0	1	0	0	0	0	1	1	1	1	12
	CaMPK7	0	1	0	0	0	0	1	0	0	0	1	1	1	0	0	0	3	1	0	0	0	9
	CaMPK9-1	2	1	0	1	0	0	0	0	0	0	0	0	2	0	0	0	1	4	0	0	0	11
	CaMPK9-2	1	2	0	1	0	0	0	0	0	0	1	0	0	0	0	0	0	1	2	0	0	8
	CaMPK13-1	0	1	0	2	0	0	1	0	0	0	1	0	1	0	0	0	1	1	0	2	1	11
	CaMPK13-2	1	5	0	2	0	0	1	0	0	0	3	0	1	0	0	0	0	1	0	1	0	15
	CaMPK17-1	0	1	0	1	0	0	0	2	0	0	0	0	1	0	1	0	0	1	0	0	0	7
	CaMPK17-2	0	2	0	0	0	0	1	1	0	0	2	0	2	0	1	0	2	3	0	0	0	14
	CaMPK16	0	5	0	2	0	0	1	0	0	0	0	0	3	0	1	0	0	1	0	0	0	13
	CaMPK19-1	3	1	0	0	0	0	0	1	0	0	0	0	2	0	0	0	2	2	0	0	0	11
	CaMPK19-2	0	0	1	0	0	0	0	1	0	1	4	0	0	0	0	1	2	3	0	0	0	13
	CaMPK20-1	0	0	0	1	0	0	1	0	0	0	3	1	0	0	0	0	3	2	0	0	0	11
	CaMPK20-2	0	0	0	2	0	0	0	0	0	1	1	0	2	0	0	0	0	1	1	1	1	10
MAPKKs	CaMKK2	2	0	0	0	0	0	0	0	0	0	1	0	3	0	0	0	0	1	0	1	0	8
	CaMKK3	0	0	0	2	0	0	1	0	0	0	4	0	0	0	1	0	1	2	0	0	0	11
	CaMKK5	0	0	0	0	1	0	1	1	0	0	1	0	0	0	0	0	1	1	1	0	0	7
	CaMKK6	0	0	0	0	0	0	1	0	0	0	1	0	1	0	0	0	3	0	0	0	0	6
	CaMKK9	1	0	0	1	0	0	0	2	0	0	2	0	0	1	0	0	0	0	1	1	0	9

### MAPK and MAPKK transcriptional expression in different organs of pepper plants

**Figure 8:**
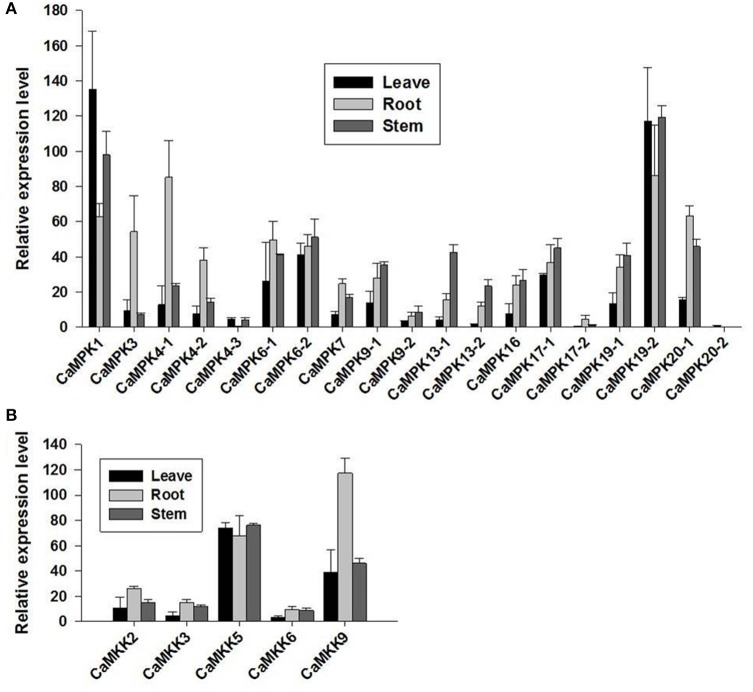
**Expression levels of MAPKs and MAPKKs in different tissues in CM334 pepper plants (leave, stem, and root)**. The expression profiles of **(A)** MAPKs and **(B)** MAPKKs in different tissues of pepper plants. The pepper plant RNA-seq data of the two gene families were obtained from a previous study.

The possible involvement of the MAPK modules in plant growth and development may be reflected in their expression patterns in different organs in pepper plants. To address this, the transcriptional expression profiles of the MAPKs and MAPKKs in different organs were analyzed to confirm their involvement in pepper development and growth using the published RNA-seq data of 6-week-old seedlings of a pepper variety (CM334), which were grown in a greenhouse on a 16-h light/8-h dark cycle (27°C/19°C). The majority of MAPK and MAPKK members expressed constitutively in leaves, roots, and stems (Figure 8). In leaves, the transcript level of *CaMPK1* was the highest, followed by *CaMPK19-2* and *CaMPK6-2*. In contrast, in roots, the transcript level of *CaMPK19-2* was among the highest, followed by *CaMPK4-1* and *CaMPK20-1*. In stems, *CaMPK19-2* showed the highest transcript level, followed by *CaMPK1* and *CaMPK6-2*. Among the five *MAPKK genes, CaMKK5* exhibited the highest transcript levels in leaf and stem, whereas *CaMKK9* exhibited the highest transcript level in roots. Taken together, these results indicate that MAPKs and MAPKKs are possibly involved in the growth or development in the tested pepper organs.

### Expression pattern analysis of MAPKS and MAPKKS in response to phytohormones, biotic, and abiotic stresses

**Figure 9:**
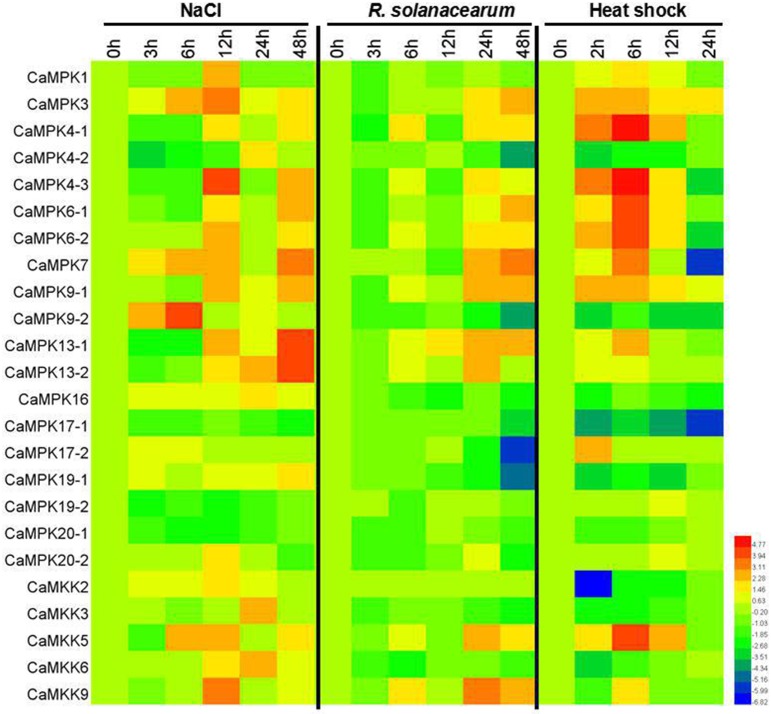
**Heat maps showing the expression patterns of the MAPKs and MAPKKs in pepper in response to R.S (***Ralstonia salanacearum***) inoculation, high salinity (200 mM NaCl) and heat shock stresses (42°C)**. Quantitative RT-PCR (qPCR) was used to represent the transcript levels of MAPKs and MAPKKs in total RNA samples extracted from pepper leaves under the treatments of RS, high salinity and heat shock stresses. The relative expression (the mean fold changed between treated and control samples at each time points ± standard deviations) was log2 transformed. Each value represents the mean relative level of expression of three replicates. Blue represents low expression, red represents high expression. The heat map was generated using the software HemI.1.0.1.

**Figure 10:**
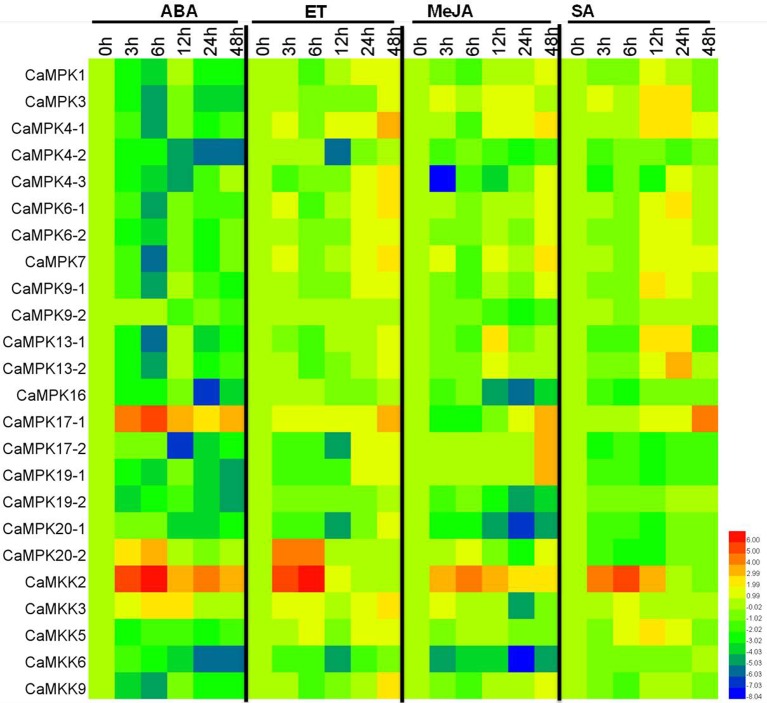
**Heat maps showing the expression patterns of the MAPKs and MAPKKs in pepper in response to phytohormones**. The expression patterns of MAPKs and MAPKKs in response to ABA (100 μM), ETH (100 μM), MeJA (100 μM), and SA (1 mM). The relative expression (the mean fold changed between treated and control samples at each time points ± standard deviations) was log2 transformed. Each value represents the mean relative level of expression of three replicates. Blue represents low expression, red represents high expression. The heat map was generated using the software HemI.1.0.1.

MAPK modules have been implicated in plant responses to biotic and abiotic stresses, and the presence of stress-responsive *cis*-elements in the promoters of the MAPK and MAPKK genes suggest their involvement in pepper response to different stresses. To confirm this speculation, the transcriptional expression of the 19 MAPKs and five MAPKKs were analyzed in pepper plants by qPCR after application of salt stress, heat shock, and RS inoculation as well as exogenous application of SA, MeJA, ETH, and ABA (Figures 9, 10 and Figures S2–S8). Almost all of the MAPKK genes exhibited modified transcriptional expression upon these challenges, and were synergistically upregulated or downregulated by two of three stresses. Similarly, the expressions of almost all 19 MAPK genes were modified differently in level and timing by the three stresses and the four exogenously applied hormones, with the majority being upregulated and a few being downregulated. For the RS inoculation, the transcript levels of *CaMPK1, CaMPK3, CaMPK4-1, 4-2, 4-3, 6-1, 6-2, 7, 9-1, 13-1, 13-2*, and *17-1* were upregulated, however, the other MAPK members exhibited downregulated, except for *CaMPK9-2* (no significant change). A total of 14 MAPK genes (*CaMPK1, CaMPK3, CaMPK4-1, 4-3, 6-1, 6-2, 7, 9-1, 13-1, 13-2, 17-1, 17-2, 19-1*, and *20-2*) were significant upregulated and 4 MAPK genes were downregulated under the heat shock stress. Under the treatment of high salinity, mRNA level of 15 MAPK genes (*CaMPK1, CaMPK3, CaMPK4-1, 4-3, 6-1, 6-2, 7, 9-1, 13-1, 13-2, 17-1, 17-2, 19-1, 20-1*, and *20-2*) exhibited significant upregulated and 4 MAPK genes were downregulated. The transcript levels of 11 MAPK genes (*CaMPK1, CaMPK3, CaMPK4-1, 4-3, 6-1, 6-2, 7, 9-1, 13-1, 13-2*, and *20-2*) and 13 MAPK genes (*CaMPK1, CaMPK3, CaMPK4-1, 4-3, 6-1, 6-2, 7, 9-1, 13-1, 13-2, 17-2, 19-2*, and *20-2*) can be significantly induced in response to exogenous application of SA and MeJA, respectively. Additionally, under the treatments of ET, the transcript levels of 16 MAPK genes (*CaMPK1, CaMPK3, CaMPK4-1, 4-2, 4-3, 6-1, 6-2, 7, 9-1, 9-2, 13-1, 13-2, 17-2, 16, 19-2*, and *20-2*) can be significant induced, and the other 3 members showed significant downregulated. Only 2 MAPK genes (*CaMPK9-*1 and *CaMPK17-1*) can be significantly induced in exposure to ABA, but the other 17 members exhibited significant downregulated.

### The interaction pattern identification of MAPKS and MAPKKS in pepper plant

**Figure 11:**
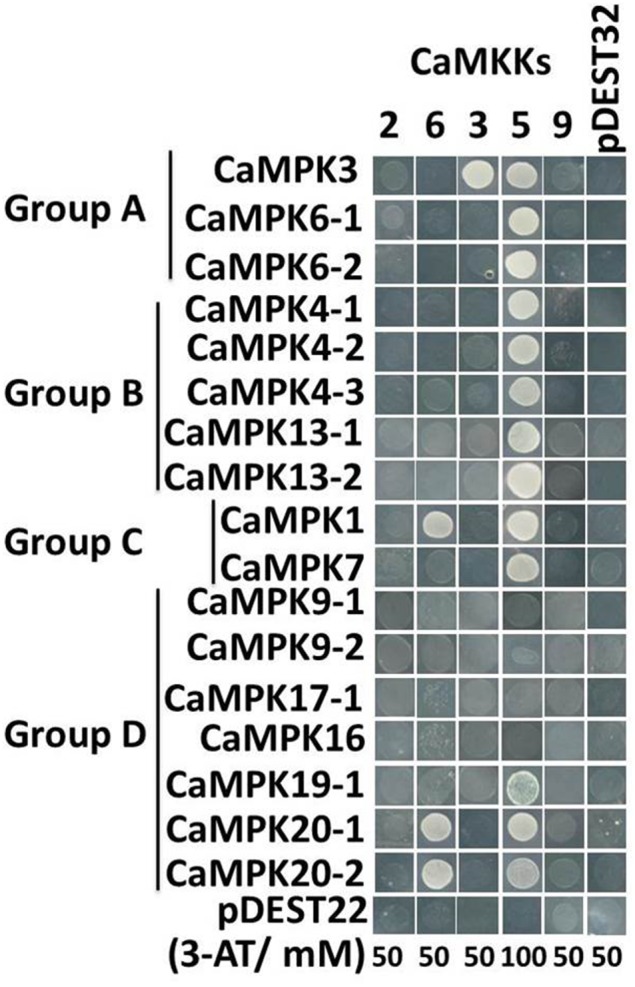
**Yeast two-hybrid (Y2H) analysis of MAPK-MAPKK interactions**. Positive interactions are indicated by yeast growth on SD-His/-Ade selection media. The indicated concentrations of 3-amino-1,2,4-triazole (3-AT), were used to suppress the auto-activation background in the Y2H system. The pepper MAPKs are divided into 4 subgroups (A–D) on the basis of their phylogenetic tree (see Figure 3). The interaction of pDEST22 and pDEST32 was used as negative control.

It's well-known that the hierarchically organized MAP Kinase kinase kinase (MAPKKK)-MAP Kinase kinase (MAPKK)-MAP kinase, all encoded by multiple genes (Smékalová et al., 2014) constitute MAPK signaling cascades, in which MAPKs serve as substrates to MAPKK, and MAPKKs serve as substrates to MAPKKKs. The MAPK cascades play important roles in different plant biological processes ubiquitously. To determine MAPKK-MAPK patterns in pepper, we systematically assessed the interactions between all the MAPKs and MAPKKs using yeast two hybrid assay except for CaMPK17-2 and CaMPK19-2 that failed to be cloned possibly due to their low transcript levels (Figure 11). The positive interactions were first selected in the synthetic SD medium lacking leucine and tryptophan and the culture was transferred to the selection medium without Leucine, tryptophan, histidine and adenine with appropriate 3-amino-1,2,4-triazole. By this method, we got interacting MAPKs of only three of the five MAPKKs, (CaMKK3, CaMKK5, and CaMKK6) while no interacting MAPK was found using CaMKK2 and CaMKK9 as baits. Among three MAPKKs that we got interacting proteins, CaMKK3 interacted with CaMPK3; CaMKK5 interacted with CaMPK3, CaMPK6-1, CaMPK6-2, CaMPK4-1, CaMPK4-2, CaMPK4-3, CaMPK1, CaMPK7, CaMPK20-1, and CaMPK20-2; CaMKK6. To test if CaMKK5 can be autoactivated by itself, we performed another round of yeast two hybrid assay with CaMKK5 inserted into both of the prey and the bait vectors, the result showed that no positive clone grew on selection SD medium (Figure S9), suggesting that CaMKK5 can not be autoactivated by itself. The interaction of PcINF1 and SRC2-1 was used as a positive control (Liu et al., 2015).

## Discussion

MAPK modules have been implicated in plant growth, development, and adaption to environmental stresses. Recently, genome-wide analyses of gene families of MAPKs, MAPKKs, and MAPKKKs have been performed in several plant species; these analyses have provided insights into the mechanisms underlying the regulation of MAPK modules, laying an important foundation for further functional characterization (Liu et al., 2010; Yin et al., 2013; Zhang et al., 2013; Wu et al., 2014). The most noticeable features of identified MAPK and MAPKK families are conservation of their main motifs, classification into four conserved subgroups, and similar intron-exon organizations within the same families and across plant species (Chen et al., 2012; Zhang et al., 2013).

### Evolution of pepper MAPKS and MAPKKS genes

In the present study, a total of 19 MAPKs and five MAPKKs were identified in the genome of the vegetable pepper, a typical Solanaceae with global agricultural and economic importance. These numbers were similar to the numbers of MAPKs and MAPKKs identified in other plant species, such as Arabidopsis (20/10), Oryza (17/8), Populus (21/11), tomato (16/5), apple (19/7), Medicago (18/4); (Ovečka et al., 2014), Phaseolus (15/9); (Neupane et al., 2013a), and soybean (38/11); (Neupane et al., 2013b). Consistent with previous results from other plant species, the 19 MAPKs in pepper possessed 11 conserved motifs, including the TEY, TDY, and MEY (only found in one MAPK) domains. The members of the MAPK and MAPKK gene families were both classified into four subfamilies, and the members within each family exhibited similar intron-exon structures. These data, together with previous studies, suggest similar origins and evolution patterns of the MAPK and MAPKK genes in different species. Possibly, the original family member duplicated prior to eudicot-monocot divergence (Zhang et al., 2013). Despite the sequence conservation, significant differences were found in the MAPK and MAPKK gene families in pepper compared to other plant species. Like tomato, pepper also possessed five MAPKKs, but this number is much less than that in Arabidopsis (10), rice (8), soybean (11), poplar (11), and *Brachypodium distachyon* (12). Although the 19 MAPK genes in pepper were similar to the 20 members in Arabidopsis, only a fraction of the MAPK genes in pepper have orthologs in Arabidopsis. In addition, several MAPK genes in Arabidopsis have more than one ortholog in the pepper genome. Perhaps, after the duplication of the origins of MAPK and MAPKK genes prior to eudicot-monocot divergence, some of the genes may have been lost during the evolution of pepper, while others might have duplicated along with the pepper genome expansion by rapid amplifications of retrotransposon elements (Kim et al., 2014). In fact, more than 81% of the pepper genome is composed of different transposable elements (TEs); (Kim et al., 2014). Additionally, differences in the sequences of MAPK and MAPKK orthologs among different plant species were also found in our study. Pepper shared much higher sequence similarities of MAPKs and MAPKKs with tomato than with Arabidopsis or rice, but the sequence similarities of MAPK and MAPKK genes in pepper to their orthologs in Arabidopsis were not always higher than to the corresponding orthologs in rice (For example, the sequence similarity between CaMPK1 and OsMPK7 is higher than that between CaMPK1 and AtMPK1), one explanation might be that some orthologs evolved slowly in the background of rice after eudicot-monocot divergence. Members of the MAPK and MAPKK families shared different amounts of sequence similarity to other plant species, indicating an imbalance in the evolutionary rates of different members of MAPK and MAPKK families, as some family members evolved more quickly than others. Furthermore, the sequences of six MAPKs were found to be different among the three pepper cultivars. Interestingly, these MAPK genes do not belong to the so-called quickly evolved genes, suggesting that mutation or evolution rates of these genes differ in different plant lineages. Further functional characterization of these divergent MAPKs in different pepper varieties might provide new insight into the mechanism underlying the regulation of MAPK modules in plant growth, development, and response to environmental stresses.

### Expression patterns of MAPKS and MAPKKS genes in pepper tissues and in response to biotic and abiotic stresses and exogenously applied hormones

Previous studies have established that plant's response to environment stress are largely regulated at the transcriptional level (Eulgem et al., 2004; Narsai et al., 2010), and the genes that transcriptionally modified against challenge of stress may play important roles in plant response to stress (Ramonell et al., 2005; Bartsch et al., 2006; Knoth et al., 2007). The RNA-seq data showed that the expression levels of the majority of pepper MAPKs and MAPKKs are relatively lower in leave, and higher in root and stem, similar observations that the majority of MAPK and MAPKK genes express preferentially in different tissues and developmental stages were also generally found (Zhang et al., 2013; Asif et al., 2014; Wei et al., 2014; Wu et al., 2014). These constitutively transcriptional expressions of MAPK and MAPKK genes suggest their possible involvement in the biological processes within the organs tested, but the precise roles and biological functions of their unequal expression levels remain to be elucidated in the future.

Under the challenges of heat shock, salt stress or RS inoculation, the majority of the MAPKs and MAPKKs in pepper genome were transcriptionally modified significantly with different timings and magnitudes in the present study. For example, within the 12 RS inoculation responsive MAPKs, *CaMPK3, CaMPK4-1, CaMPK6-1, CaMPK6-2, CaMPK7, CaMPK9-1*, and *CaMPK13-1* were transciptionally upregulated by RS inoculation mainly at later time points, while *CaMPK4-2, CaMPK17-1* and *CaMPK19-1* were transcriptionally upregulated by RS inoculation at 3 and 6 h post inoculation. These results are consistent with that of the previous studies that MAPK and MAPKK genes are transcriptionally modified by various biotic or abiotic stresses (Liu et al., 2010; Chen et al., 2012; Yin et al., 2013; Zhang et al., 2013), for example, *SpMPK3* (Li et al., 2014), MAP kinase 4 of barley (Abass and Morris, 2013), and *GhMPK2* (Zhang et al., 2011a,b) were found to be involved in plant response to abiotic and biotic stresses. All these results indicate that MAPKs or MAPKKs in plants mainly act as positive regulators, only a small number act as negative regulators in response to biotic and abiotic stresses. Similar observations have been made in genomes of other plant species such as *Brassica rapa* (Lu et al., 2015), cucumber (Wang et al., 2015), *Brachypodium distachyon* (Jiang et al., 2015), maize (Sun et al., 2015), grapevine (Wang et al., 2014a), and tomato (Wu et al., 2014). By previous studies, a group of MAPK or MAPKK families' members may be required in the fine tune of plant response to a specific stress. For example, MPK9 and MPK12 (Jammes et al., 2011), MPK3, MPK4, and MPK6 (Pitzschke et al., 2009) in Arabidopsis function coordinately in defense reaction. However, the precise roles of these genes and how they coordinate in the response of plant to specific stress remain to be determined. In addition, despite the presence of genes specifically responding to a specific stress, members in MAPK and MAPKK families responding to two or three stresses were also found in the pepper genome in the present study, for example, *CaMPK7* was synergistically induced by RS inoculation, heat stress and NaCl treatment, *CaMPK4-1* was induced by heat stress, but was down-regulated by both RS inoculation and salinity, and *CaMPK9-*1 was upregulated by salt stress and RS inoculation, suggesting a diverse patterns of crosstalk between the tested three stresses mediated by different MAPK signaling modules. Consistently, the majority of mulberry MAPK family members were previously found to respond to different stresses (high/low temperature, salt, and drought); (Wei et al., 2014), and MAPKs or MAPKKs such as ZmMPK17 (Pan et al., 2012), GhMPK6a (Li et al., 2013), ZmMAPK1 (Wu et al., 2015), ZmSIMK1 (Wang et al., 2014b), ZmMKK1 (Cai et al., 2014), ZmMPK5 (Zhang et al., 2014) were also found to be involved in plant response to multiple stresses by individual studies, and some MAPKs were found to act as molecular linkers between abiotic stress signaling and plant reproduction(Sun et al., 2015). We speculate that MAPKs and MAPKKs might act as crucial nodes in crosstalk between plant response to biotic and abiotic stresses, which provide great potential in the fine tune of plant response to different stresses (Fujita et al., 2006; Schenke et al., 2011).

Since hormones such as SA, JA, ET, and ABA act as second messages ubiquitously in the response of plant to abiotic and biotic stresses (Seyfferth and Tsuda, 2014), and have been previously implicated in plant immunity, plant response to heat shock, salt stress, and pathogen infections, and crosstalk between plant response to biotic and abiotic stresses (Fujita et al., 2006; Clarke et al., 2009; Chan, 2012; van Zanten et al., 2012; Dang et al., 2014; Derksen et al., 2013; Hou et al., 2013; O'Brien and Benková, 2013; Singh and Jwa, 2013). In addition, MAPK cascades have been generally found to interact with signaling pathways mediated by hormones such as SA, JA, and ET (Smékalová et al., 2014), and hormone responsive-elements such as JA-responsive MeJA (Beaudoin and Rothstein, 1997), ethylene responsive-ERE (Itzhaki et al., 1994), and GCC-box (Ohme-Takagi and Shinshi, 1995), salicylic acid-responsive-TCA element (Goldsbrough et al., 1993), and abscisic acid-responsive ABRE (Guiltinan et al., 1990) were extensively found in the promoters of MAPK and MAPKK genes of pepper. To test the possible interactions between MAPK signaling in pepper and pathways mediated by hormones, the transcriptional modification of MAPKs and MAPKKs were determined by real-time PCR against the exogenous application of SA, MeJA, ETH, and ABA. Similarly to that challenged with the aforementioned abiotic stresses and RS inoculation, the majority of MAPKs and MAPKKs were found to be transcriptionally modified significantly by exogenously applied SA, MeJA, ETH, and ABA, and we got the similar results as the previous studies (Shin et al., 2001; Huh et al., 2015) that *CaMPK3* (named as *CaMK1* in the previous study) and *CaMPK6-1* (named as *CaMK2* in the previous study) can be induced in response to RS inoculation, high salinity and heat shock stresses, as well as exogenously applied SA, MeJA, ET, and ABA. We speculate that transcriptional modification of MAPK and MAPKK genes by heat shock, salt stress, and RS inoculation might correlate to signaling pathways mediated by SA, JA, ET, and ABA. Moreover, the MAPK modules may also act as components in hormone signal transduction (Singh and Jwa, 2013), and some of the MAPKs or MAPKKs responding to more than one exogenously applied hormones may act as convergent nodes in the integration of upstream signaling and in the orchestration of multiple cellular processes.

### Patterns of pepper MAPK and MAPKK interactions and their possible roles in response of pepper to stresses

MAPK cascades, which consist of many distinct MAPKKK-MAPKK-MAPK modules linked to various upstream receptors and downstream targets through sequential phosphorylation and activation of the cascade components, are involved in different biological processes. Despite the occurrence of a large number of possible MAPK modules, no MAPK module has been experimentally identified in pepper so far. Yeast two hybrid system was generally employed to determine the MAPK interactome (Singh et al., 2012; Kong et al., 2013b). The MAPKK-MAPK interactome of pepper was assayed in this study, and the results showed that only CaMKK3, CaMKK5, and CaMKK6 got interacting MAPKs, one possibility may be that the other CaMKK2 and CaMKK9 might interacting with CaMPK17-2 and CaMPK19-2, which failed to be cloned possibly due to their low transcript level, another possibility can not be excluded that interacting MAPKs may not be completely found with the unphosphorylated MAPKKs as bait under the yeast two hybrid system, similarly to the results in maize (Kong et al., 2013b). Similarly to the previous studies in rice (Ding et al., 2009; Singh et al., 2012), our result in the present study showed that one MAPKKs may interact with multiple MAPKs. For example, the heat stress inducible CaMKK5 interacts significantly with CaMPK3, CaMPK6-1, CaMPK6-2, CaMPK4-1, CaMPK4-2, CaMPK4-3, CaMPK1, CaMPK7, CaMPK20-1, and CaMPK20-2. Similarly, CaMKK6 interacts significantly with CaMPK1, CaMPK20-1, and CaMPK20-2. Conversely, a single MAPK can interact with multiple upstream MAPKKs, for example, CaMPK1, CaMPK20-1 and CaMPK20-2 each interact with two MAPKKs, indicating that the MAPKK-MAPK modules constitute signaling networks. Our data also showed that the interaction of CaMKK5 to multiple MAPKs is not due to its autoactivation, to elucidate the underlying mechanism of the interactions of unphosphorylated MAPKKs, for example CaMKK5, with multiple MAPKs, further investigations are needed. Since both MAPKKs and MAPKs in pepper were upregulated or downregulated transcriptionally by heat, salt stress and RS inoculation as well as by exogenously applied SA, MeJA, ETH, and ABA with different timing, magnitude, these modules may collaborate in transmitting upstream signals into appropriate downstream cellular responses and processes.

Collectively, a total of 19 pepper MAPK genes and five MAPKK genes were identified in the present study. Compared to MAPK and MAPKK families in other plant species, comparable conservations and significant divergences among pepper and other plant species were found in the MAPK and MAPKK gene families. The majority of MAPKK and MAPK genes were expressed preferentially in all tested organs and were also transcriptionally modified in leaves by heat shock, salt stress, and RS inoculation, as well as the exogenous application of SA, MeJA, ETH, and ABA. By real time PCR, one specific stimulus simultaneously modified the expression of several MAPKs and MAPKKs, and one MAPK or MAPKK gene might respond synergistically to several stimuli. In addition, by interactions, MAPKKs and MAPKs constitute modules and signaling network, which might have important roles in orchestrating pepper growth, development, and response to specific biotic or abiotic stress. These results will facilitate the future functional characterization of MAPK cascades in pepper.

## Author contributions

Conceived and designed the experiments: ZL and SH. Performed the experiments: ZL, LS, YL, LS, JC, QT, and JW. Analyzed the data: ZL, SY, XH, YL, YL, JH, CL, SM, and DG. Wrote the paper: ZL and SH. Provided guidance on the whole study: ZL and SH.

## Conflict of interest statement

The authors declare that the research was conducted in the absence of any commercial or financial relationships that could be construed as a potential conflict of interest.

## Supplementary material

Table S1 **Nomenclature for MAPKs and MAPKKs in Arabidopsis, tomato, rice, and soybean**.

Table S2 **Primers used in this study and the qPCR validation**.

Figure S1 **Multiple sequence alignment of the amino acid sequences of pepper MAPKs in three different pepper cultivars**.

Figure S2 **The expression pattern of the MAPKs and MAPKKs in pepper in response to NaCl**. **(A)** The expression pattern of MAPKs in response to NaCl. **(B)** The expression pattern of MAPKKs in response to NaCl. Asterisks indicate a significant difference as determined by Fisher's protected LSD test (*P* < 0.05).

Figure S3 **The expression pattern of the MAPKs and MAPKKs in pepper in response to heat shock**. **(A)** The expression pattern of MAPKs in response to heat shock. **(B)** The expression pattern of MAPKKs in response to heat shock. Asterisks indicate a significant difference as determined by Fisher's protected LSD test (*P* < 0.05).

Figure S4 **The expression pattern of the MAPKs and MAPKKs in pepper in response to pathogen infection**. **(A)** The expression pattern of MAPKs in response to pathogen infection. **(B)** The expression pattern of MAPKKs in response to pathogen infection. Asterisks indicate a significant difference as determined by Fisher's protected LSD test (*P* < 0.05).

Figure S5 **The expression pattern of the MAPKs and MAPKKs in pepper in response to exogenous SA**. **(A)** The expression pattern of MAPKs in response to exogenous SA. **(B)** The expression pattern of MAPKKs in response to exogenous SA. Asterisks indicate a significant difference as determined by Fisher's protected LSD test (*P* < 0.05).

Figure S6 **The expression pattern of the MAPKs and MAPKKs in pepper in response to exogenous MeJA**. **(A)** The expression pattern of MAPKs in response to exogenous MeJA. **(B)** The expression pattern of MAPKKs in response to exogenous MeJA. Asterisks indicate a significant difference as determined by Fisher's protected LSD test (*P* < 0.05).

Figure S7 **The expression pattern of the MAPKs and MAPKKs in pepper in response to exogenous ET**. **(A)** The expression pattern of MAPKs in response to exogenous ET. **(B)** The expression pattern of MAPKKs in response to exogenous ET. Asterisks indicate a significant difference as determined by Fisher's protected LSD test (*P* < 0.05).

Figure S8 **The expression pattern of the MAPKs and MAPKKs in pepper in response to exogenous ABA**. **(A)** The expression pattern of MAPKs in response to exogenously ABA. **(B)** The expression pattern of MAPKKs in response to exogenously ABA. Asterisks indicate a significant difference as determined by Fisher's protected LSD test (*P* < 0.05).

Figure S9 **The autoactivation identification of CaMKK5 and CaMKK5 by yeast two hybird**. Yeast cells were plated on SD/–Leu/–Trp/–His medium containing 100mM 3-amino-1,2,4-triazole. The interaction of PcINF1 and SRC2-1 was used as positive control.
