# Supplementary figures and images for: Genome-wide identification and transcriptional expression analysis of mitogen-activated protein kinase and mitogen-activated protein kinase kinase genes in Capsicum annuum
Source: Front Plant Sci. 2015 Sep 25;6:780. doi: 10.3389/fpls.2015.00780 (PMC4585111; doi:10.3389/fpls.2015.00780)

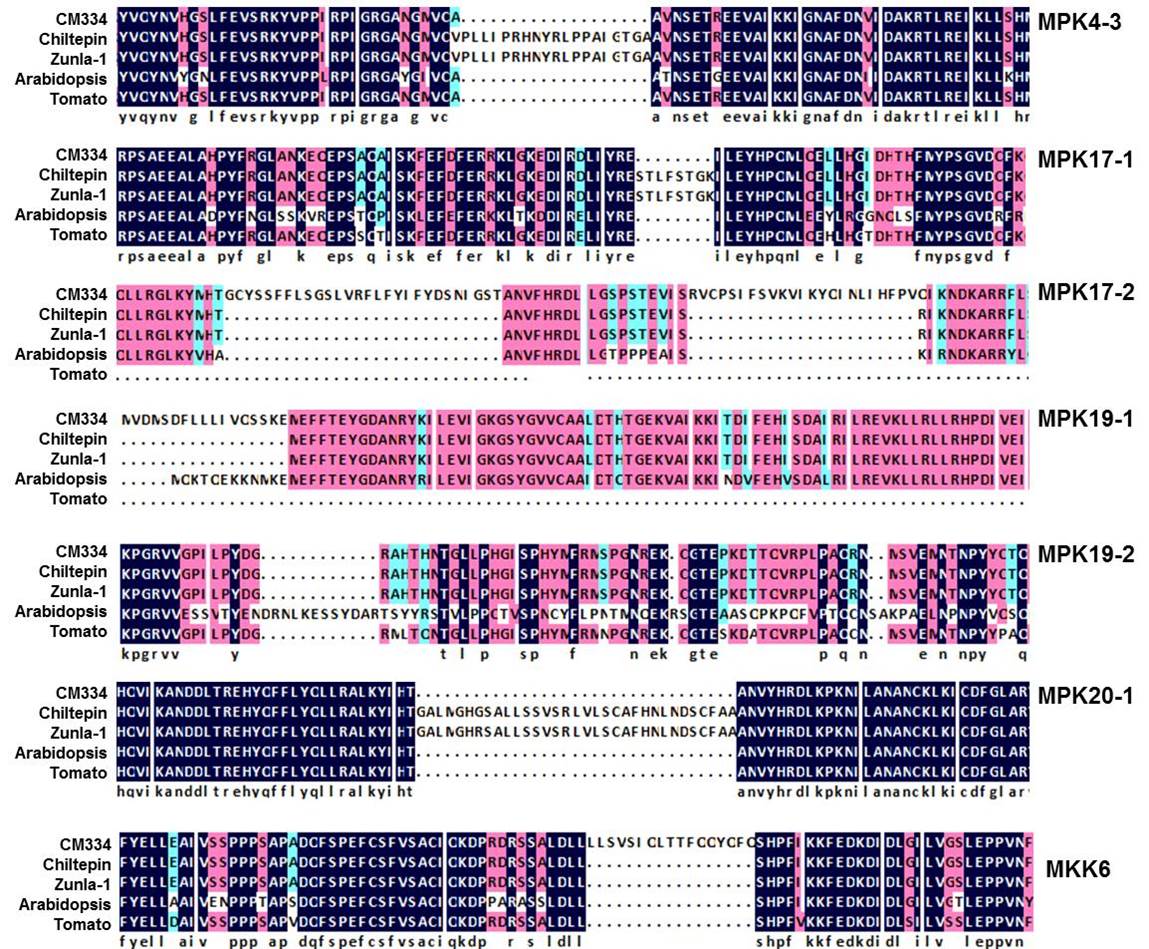

Supplement: Figure S1 — Multiple sequence alignment of the amino acid sequences of pepper MAPKs in three different pepper cultivars. [file Image1.JPEG]

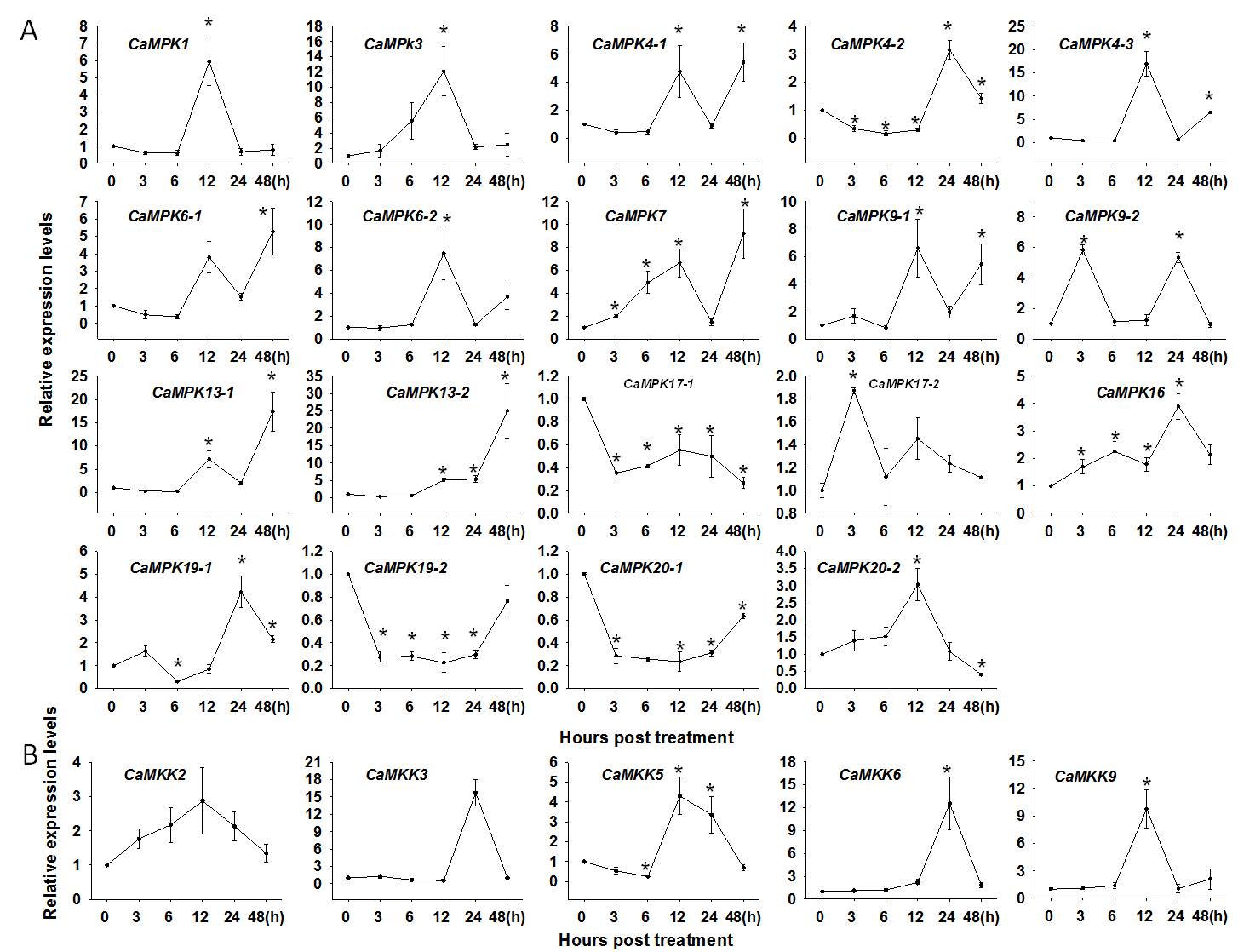

Supplement: Figure S2 — The expression pattern of the MAPKs and MAPKKs in pepper in response to NaCl. (A) The expression pattern of MAPKs in response to NaCl. (B) The expression pattern of MAPKKs in response to NaCl. Asterisks indicate a significant difference as determined by Fisher's protected LSD test (P < 0.05). [file Image2.JPEG]

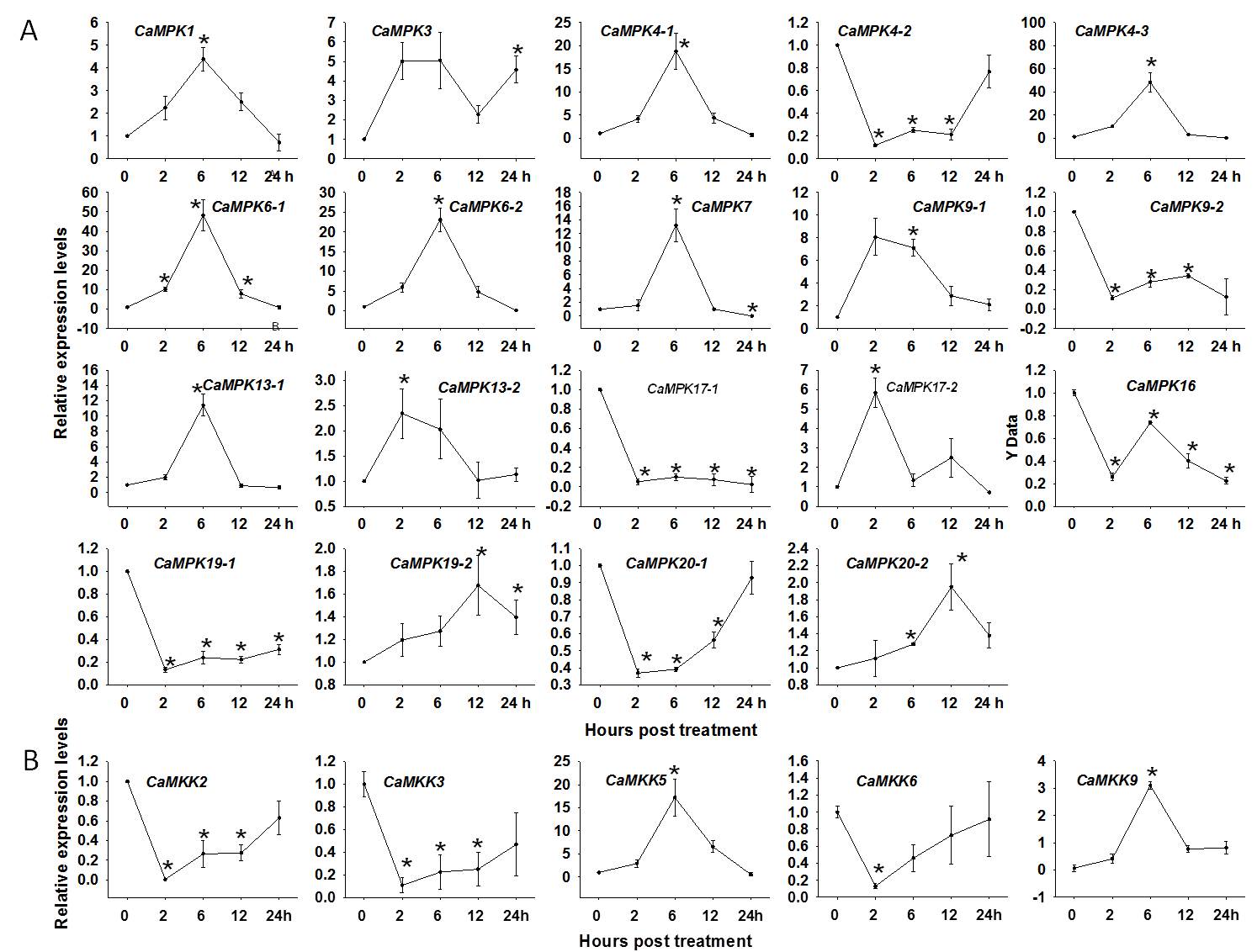

Supplement: Figure S3 — The expression pattern of the MAPKs and MAPKKs in pepper in response to heat shock. (A) The expression pattern of MAPKs in response to heat shock. (B) The expression pattern of MAPKKs in response to heat shock. Asterisks indicate a significant difference as determined by Fisher's protected LSD test (P < 0.05). [file Image3.JPEG]

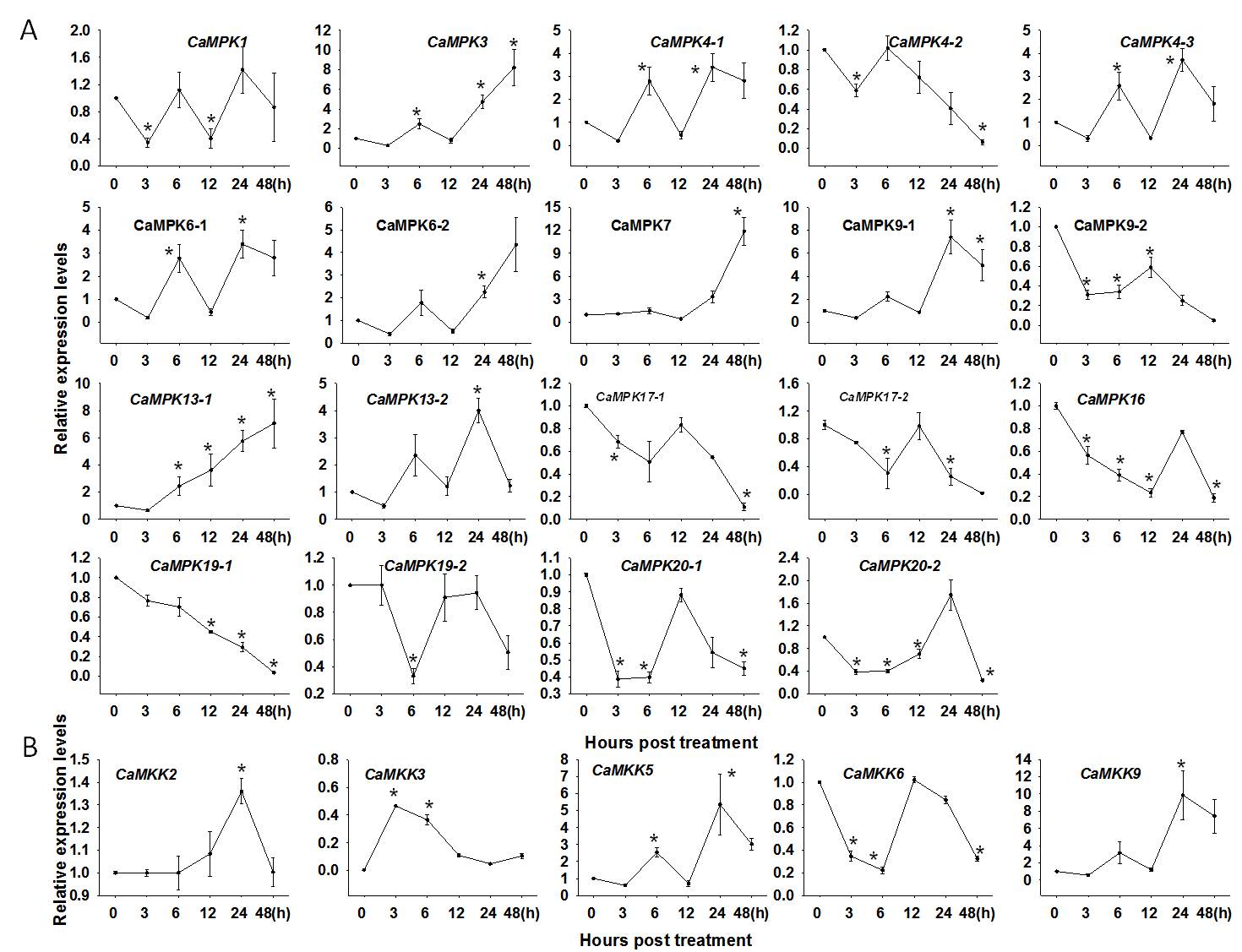

Supplement: Figure S4 — The expression pattern of the MAPKs and MAPKKs in pepper in response to pathogen infection. (A) The expression pattern of MAPKs in response to pathogen infection. (B) The expression pattern of MAPKKs in response to pathogen infection. Asterisks indicate a significant difference as determined by Fisher's protected LSD test (P < 0.05). [file Image4.JPEG]

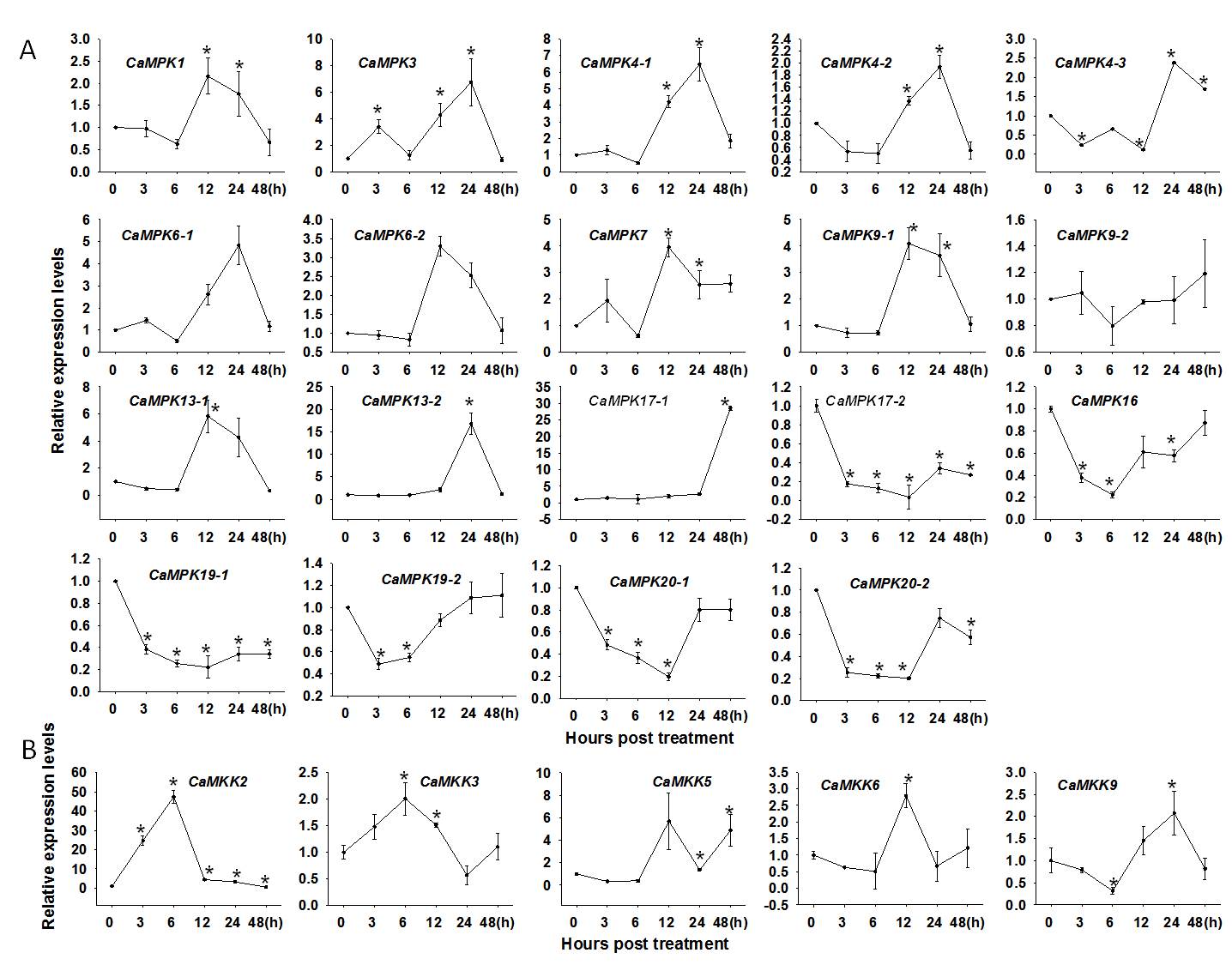

Supplement: Figure S5 — The expression pattern of the MAPKs and MAPKKs in pepper in response to exogenous SA. (A) The expression pattern of MAPKs in response to exogenous SA. (B) The expression pattern of MAPKKs in response to exogenous SA. Asterisks indicate a significant difference as determined by Fisher's protected LSD test (P < 0.05). [file Image5.JPEG]

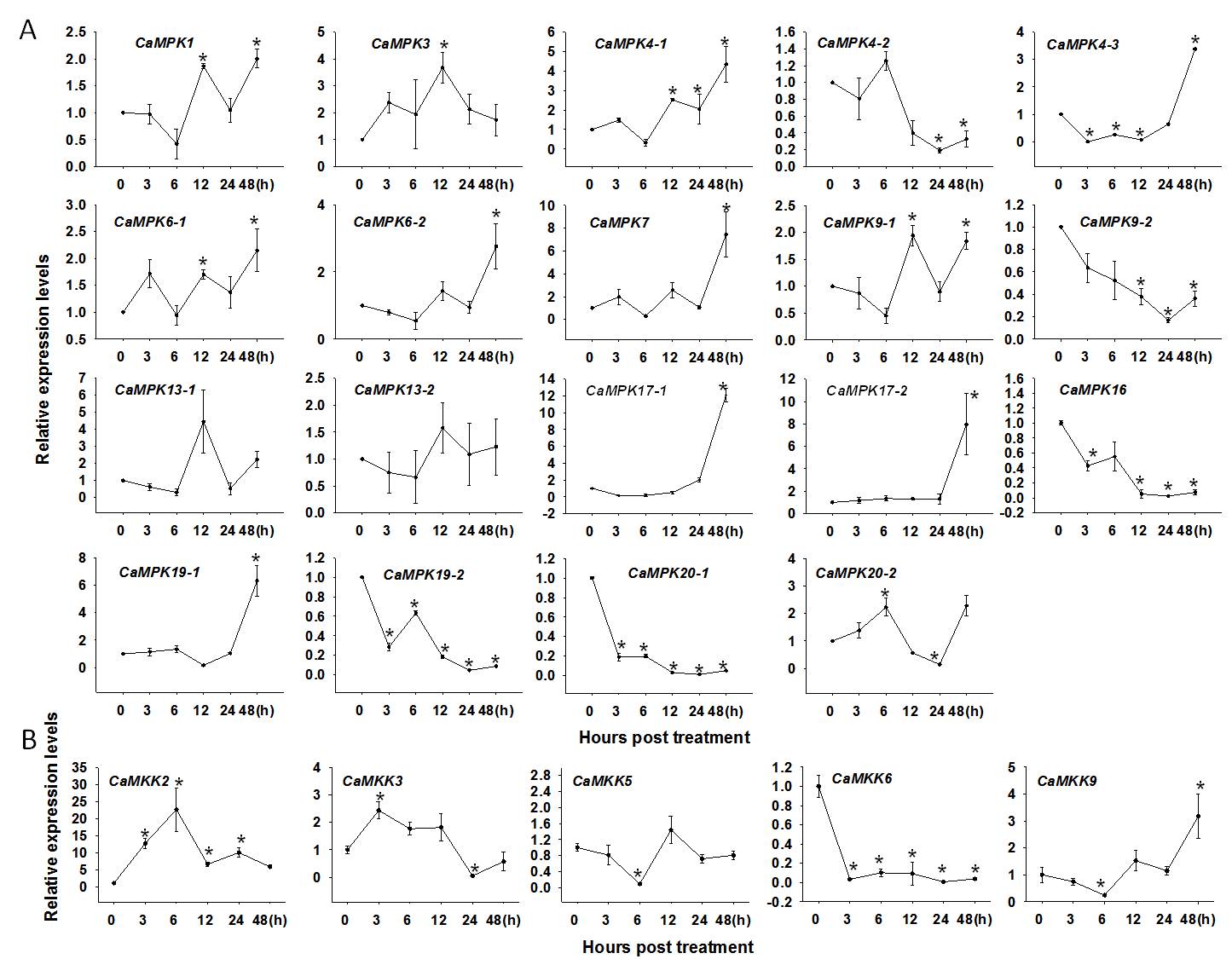

Supplement: Figure S6 — The expression pattern of the MAPKs and MAPKKs in pepper in response to exogenous MeJA. (A) The expression pattern of MAPKs in response to exogenous MeJA. (B) The expression pattern of MAPKKs in response to exogenous MeJA. Asterisks indicate a significant difference as determined by Fisher's protected LSD test (P < 0.05). [file Image6.JPEG]

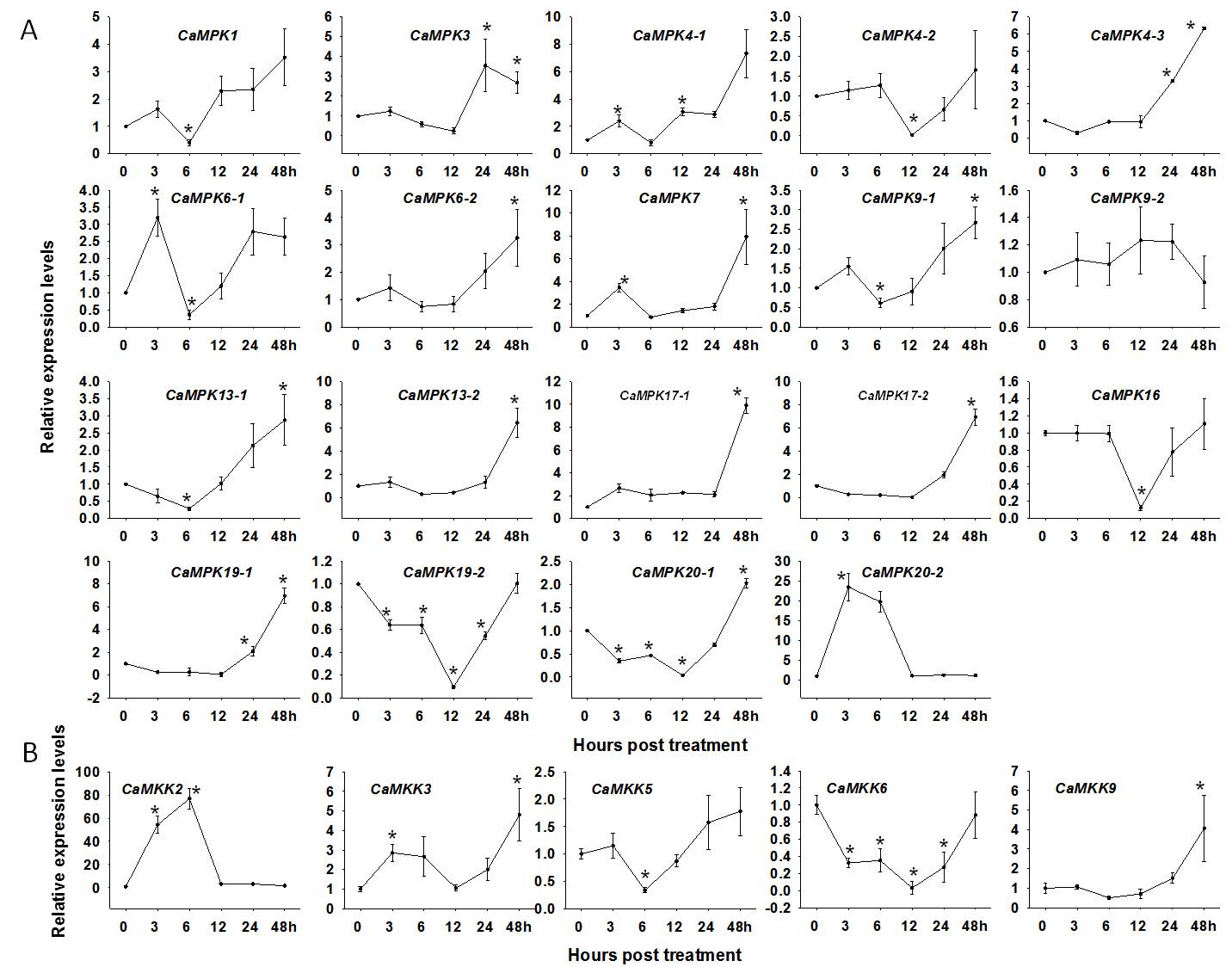

Supplement: Figure S7 — The expression pattern of the MAPKs and MAPKKs in pepper in response to exogenous ET. (A) The expression pattern of MAPKs in response to exogenous ET. (B) The expression pattern of MAPKKs in response to exogenous ET. Asterisks indicate a significant difference as determined by Fisher's protected LSD test (P < 0.05). [file Image7.JPEG]

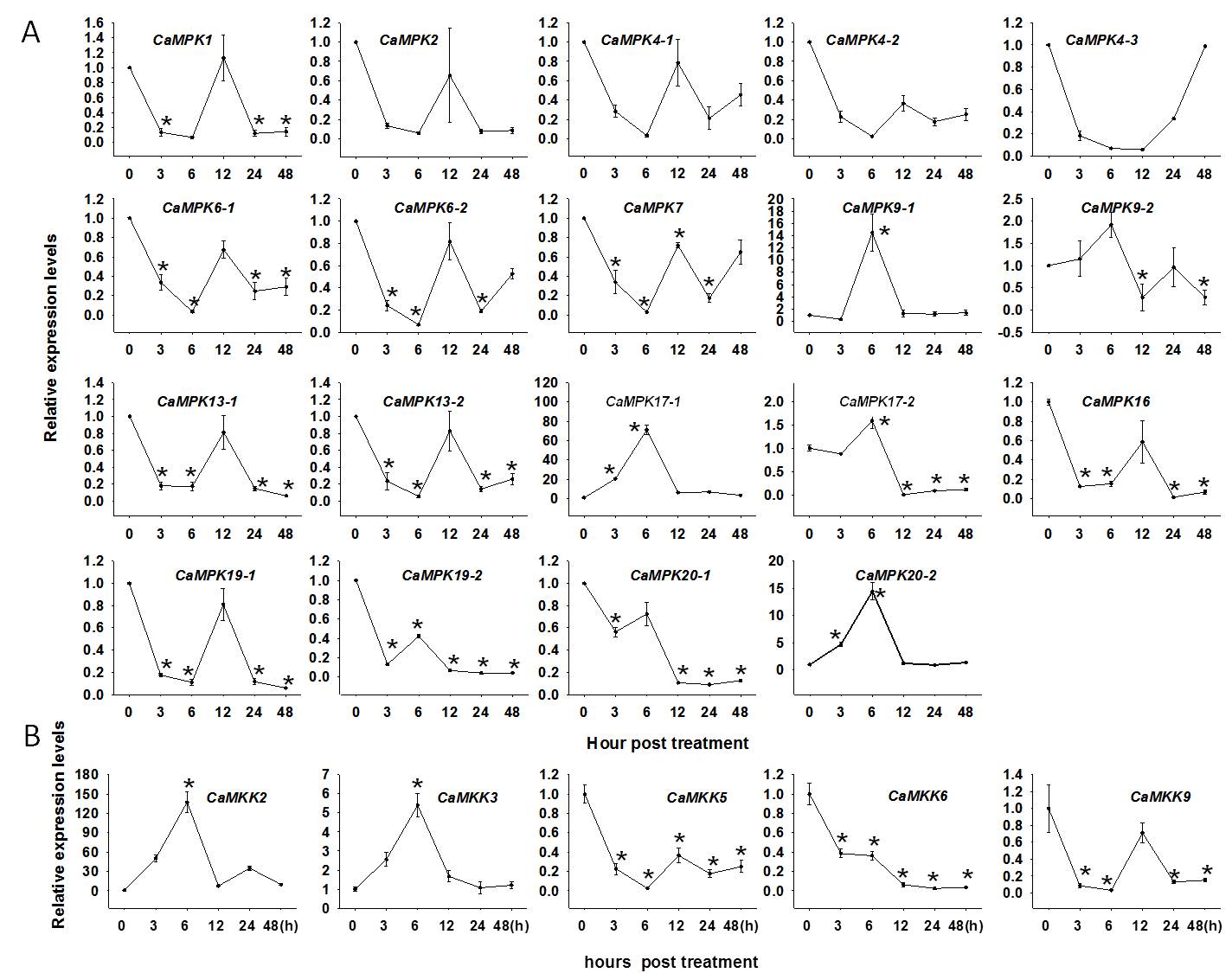

Supplement: Figure S8 — The expression pattern of the MAPKs and MAPKKs in pepper in response to exogenous ABA. (A) The expression pattern of MAPKs in response to exogenously ABA. (B) The expression pattern of MAPKKs in response to exogenously ABA. Asterisks indicate a significant difference as determined by Fisher's protected LSD test (P < 0.05). [file Image8.JPEG]

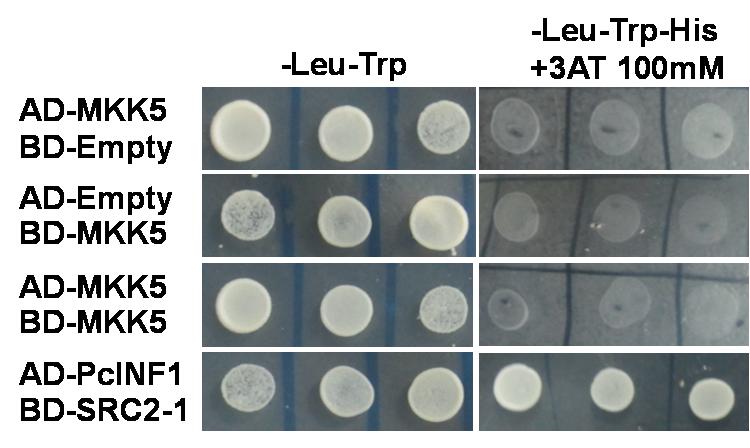

Supplement: Figure S9 — The autoactivation identification of CaMKK5 and CaMKK5 by yeast two hybird. Yeast cells were plated on SD/–Leu/–Trp/–His medium containing 100mM 3-amino-1,2,4-triazole. The interaction of PcINF1 and SRC2-1 was used as positive control. [file Image9.JPEG]
